# Profiling mRNA, miRNA and lncRNA expression changes in endothelial cells in response to increasing doses of ionizing radiation

**DOI:** 10.1038/s41598-022-24051-6

**Published:** 2022-11-19

**Authors:** Sunita Chopra, Uma Shankavaram, Michelle Bylicky, Juan Dalo, Kevin Scott, Molykutty J. Aryankalayil, C. Norman Coleman

**Affiliations:** Radiation Oncology Branch, Center for Cancer Research, National Cancer Institute (NCI), Bethesda, MD 20892 USA

**Keywords:** Predictive markers, Non-coding RNAs, Transcriptomics, Predictive markers

## Abstract

Recent and past research have highlighted the importance of the endothelium in the manifestation of radiation injury. Our primary focus is on medical triage and management following whole body or partial-body irradiation. Here we investigated the usability of endothelial cells’ radiation response for biodosimetry applications. We profiled the transcriptome in cultured human endothelial cells treated with increasing doses of X-rays. mRNA expression changes were useful 24 h and 72 h post-radiation, microRNA and lncRNA expression changes were useful 72 h after radiation. More mRNA expressions were repressed than induced while more miRNA and lncRNA expressions were induced than repressed. These novel observations imply distinct radiation responsive regulatory mechanisms for coding and non-coding transcripts. It also follows how different RNA species should be explored as biomarkers for different time-points. Radiation-responsive markers which could classify no radiation (i.e., ‘0 Gy’) and dose-differentiating markers were also predicted. IPA analysis showed growth arrest-related processes at 24 h but immune response coordination at the 72 h post-radiation. Collectively, these observations suggest that endothelial cells have a precise dose and time-dependent response to radiation. Further studies in the laboratory are examining if these differences could be captured in the extracellular vesicles released by irradiated endothelial cells.

## Introduction

Understanding the radiation response in the context of normal tissue is important in two ways. First, injury to adjacent normal tissue during cancer radiotherapy may be responsible for post-therapy complications and, second, normal tissue and life-threatening radiation injuries may occur during accidental or intentional full-body or partial-body radiation exposures from an industrial disaster or a terrorist incident. Recent research has shown that endothelial cell damage is one of the underlying causes of both early and late effects of radiation in normal tissue^1,2^. For instance, radiation-induced complications have been extensively observed in the heart and its major blood vessels^3–6^. Endothelial cell damage has been proposed to underlie these complications^7–9^ which include atherosclerosis and other cardiovascular diseases such as coronary artery disease, carotid artery disease, peripheral artery disease and ischemic stroke^10^. Endothelial cell damage was also observed in response to low-dose radiation in the mouse retinas^11^. Radiation-induced kidney toxicity included damage to endothelial cells^12^. A recent study showed radiation induced endothelial cell decrease in rat kidneys in response to 13 Gy X-rays. This vascular regression depended on increased miR-34a and repression of its target, Jagged^13^. Radiation-induced intestinal injury has also been observed to be endothelium dependent^14–16^. Fliedner et al. studied the 110 patients of accidental whole body radiation injury from 1945 to 2000^17^. They concluded that the radiation syndrome was multi-organ with vascular endothelial damage underlying all the recorded injuries^17^.

Radiation oncologists and radiation biology investigators have long been aware of the centrality of the endothelial damage in the appearance of normal tissue toxicity^2^, and attempts have been made to understand the underlying mechanisms of radiation effects on endothelium. The current mechanistic understanding of radiation effects on endothelium has been collated by Venkatesulu et al., 2018^18^ and Wijerathne et al., 2021^19^. P53 and sphingomyelin ceramide apoptotic pathways are activated in response to radiation in endothelial cells^18^. Paris et al., 2001 showed how endothelial cell apoptosis is the primary injury leading to stem cell loss and crypt erosion in radiation-induced gastrointestinal syndrome in mice^16^ and that this apoptosis was governed by acid sphingomyelinase gene and could be rescued by basic Fibroblast Growth Factor^16^. Chronic endothelial cell senescence involving PI3K/mTOR pathway and senescence associated secretory phenotype (SASP) could also be observed depending on the cell-type, cellular context, radiation dose and dose rate^18^. It was also established decades ago that different caliber blood vessels also displayed differential radiosensitivity^20^. Endothelial barrier function is compromised directly by apoptosis and indirectly by changes in trans-membrane potential differences and permeability changes^21^.

Few studies have explored the transcriptional landscape of endothelial cells in response to radiation. We have previously shown that immune and stress response pathways were dysregulated in irradiated coronary artery endothelial cells with multi-fractionated regimen eliciting stronger response compared to single dose radiation in the 24 h post irradiation^22^. Persistent changes in cell cycle and inflammation pathways in human coronary artery cells after radiation were also observed by Baselet et al^23^. Himburg et. al. found that bone marrow derived endothelial cells respond to 5 Gy radiation with an altered gene expression signature 24 h post-radiation but changes normalized by 14 days^24^. microRNA expression changes have also been previously studied where several miRNAs were found to be downregulated in response to radiation^25^. Alterations in the glycome of endothelial cells were also observed in response to radiation^26^. Several other studies have looked at transcriptional and other molecular responses of the endothelium to radiation using human umbilical vein endothelial cells^27–29^. In an elegant study published by Morilla et al. in 2021, authors dissected out multi-omics comprising miRnome, transcriptome, proteome and metabolome to identify consistent changes evolving over time in response to radiation^30^. The group identified alterations in angiogenesis in addition to DNA damage response, apoptosis, senescence, immune-response and fibrosis^30^. Radiation induced senescence signature with Interleukin-1 signaling pathway playing a key role was recently identified in cultured endothelial cells^31^. Bone marrow derived endothelial cells were investigated for transcriptional changes in response to 5 Gy total body irradiation (TBI) in mice and the group found that the effect peaked at 24 h but by 14 days post-radiation most changes had resolved^24,32^.

Our laboratory is interested in the endothelial cell response as a component of molecular-targeted adaptive radiotherapy^17,33^. We have expanded our research to the endothelium as a target for the whole-body irradiation and radiation medical countermeasures as did other groups as reported by Satyamitra from an NIAID workshop^22,34^. Our previous work explored blood and organ-based gene expression patterns correlating with total body irradiation exposures and survival in Gottingen minipigs, mouse, and non-human primates. The broader goal of our current studies is to understand how endothelial cells might contribute to the total-body radiation response in the form of circulating RNA and extra-cellular vesicles bearing radiation specific RNA signatures. In this paper, we address the first part of the study by examining radiation-induced RNA responses inherent to the endothelial cells. In a soon to be published work, we explored radiation responsive RNAs in exosomes generated by endothelial cells treated with radiation.

Finding an ideal cell-line for our studies was a challenge. Human umbilical endothelial cells make ideal candidates for conducting proof of principle and mechanistic studies but would have been a poor choice for the present work where we want to understand endothelial cell biology in TBI context. The heterogeneity in radiation response of larger vessels versus smaller microvasculature has been well commented on. Capillaries are known to be most radiation sensitive owing to presence of a single layer of endothelium, tunica intima while the larger vessels have three layers- the tunica intima, media, and adventitia^18^. Not many studies have explored the relative radiation sensitivities of endothelial cells in culture conditions from different vessel types. Comparing endothelial cells derived from different organs, Park et al., concluded that hepatic sinusoidal endothelial cells were most radioresistant while dermal endothelial cells were most radiation sensitive^35^. The aortic endothelial cells respond to radiation^36–38^ but a comprehensive look at the transcriptional changes has not yet been attempted. For our objective to investigate whether endothelial cells could provide valuable information which could lead to development of markers of radiation damage we chose to use aortic endothelial cells. Aorta is the largest vessel and hence more relevant for studies exploring the release of extra-cellular vesicles into the blood. The ease of culturing them in large numbers to collect enough RNA and extra-cellular vesicles to perform assays was another factor. We profiled gene expression, long non-coding RNA and miRNA expression at multiple doses (1 Gy, 2 Gy, 4 Gy, 8 Gy and 10 Gy) and two time-points (24 h and 72 h) in primary human aortic endothelial cells.

We hypothesize that study of interdependent molecular changes from endothelial cells would be informative for a radiation-predictive biomarker signature identification and investigation of potential targets for radiation-mitigation and prevention. A perceived limitation of the study is the use of static culture systems even though endothelial cells always experience laminar shear stress under normal physiological conditions inside the body. We are developing and employing 3-D chip-based culture systems in our lab to address such questions. These chips simulate not only laminar shear stress but also multi-cellular environment of live organs. The current study provides an underlying framework to build on in the future.

## Results

### Dose and time dependent changes observed in expression levels of mRNA, lncRNA and miRNA transcripts

**Figure 1:**
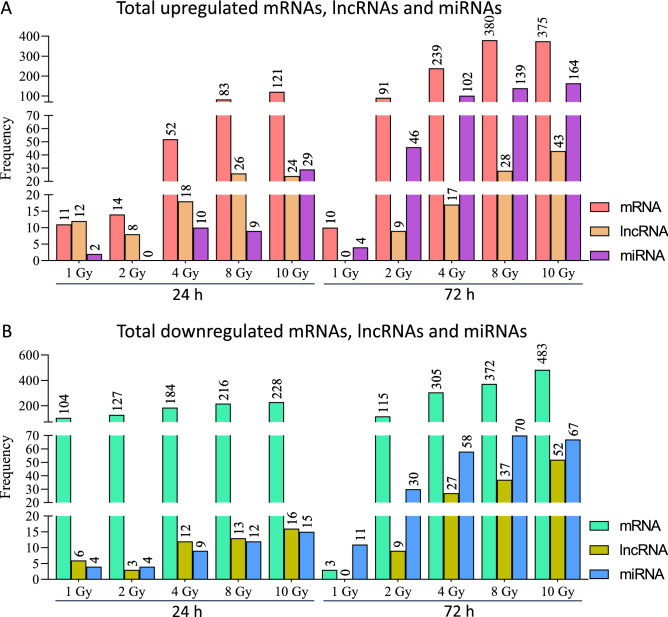
Summary of the sequencing data. (**A**) Total numbers of differentially upregulated mRNAs, lncRNAs and miRNAs for each dose and both 24 h and 72 h. (**B**) Total number of differentially downregulated mRNAs, lncRNAs and miRNAs for each dose and both 24 h and 72 h. ANOVA (FDR < 0.05) followed by Dunnett’s test (p-value < 0.05) was used to identify differentially expressed RNA molecules in each dose compared to control samples (fold change cut-off set at |2|).

Principal Component Analysis was performed on the normalized data before applying any statistical tests to identify overall differences across all samples and data-points (Supplementary Fig. 1). The 24 h and 72 h data points clustered separately as did lower (0 Gy, 1 Gy, 2 Gy) vs. medium (4 Gy) vs. higher (8 Gy, 10 Gy) dose points for mRNA and lncRNA data. The separation in miRNA data was not sharp between lower doses at 24 h but all the doses separated at 72 h which also explains the lower number of miRNAs found differentially expressed in subsequent analyses at 24 h. Differentially expressed mRNA transcripts which satisfied the cut-off criteria of fold change (> = 1.5) and an adjusted p-value (FDR < 0.05) in pair-wise comparisons of each dose with the 0 Gy control samples were selected for these results (Supplementary Fig. 2). mRNA, lncRNA and miRNA transcripts followed a dose-dependent regulation at 24 h and 72 h time points (Fig. 1). Quantitatively, mRNA transcripts had a similar response to radiation at both 24 h and 72 h time-points while more miRNA and lncRNA transcripts changed at the 72 h time-point (Fig. 1). More mRNA transcripts were downregulated than upregulated across all dose-points while more miRNA and lncRNA transcripts were upregulated than downregulated at most of the dose-points (Fig. 1). Ninety-three mRNA transcripts, two miRNA transcripts and six lncRNA transcripts were commonly regulated across all doses at the 24 h time point (Supplementary Fig. 3A,C,E). At 72 h only nine mRNA, 10 miRNA and no lncRNA transcripts were commonly regulated across all doses (Supplementary Fig. 3B,D,F). In the Venn diagrams all the quadrants with more than 10 transcripts are highlighted in yellow (Supplementary Fig. 3). Owing to smaller number of miRNA and lncRNA transcripts changing at 24 h time-point, very few dose-overlapping molecules were observed (Supplementary Fig. 3C,E). Significant overlap in the regulated mRNA transcripts between 24 and 72 h time-points at each dose separately was observed barring 1 Gy at 24 h (Supplementary Fig. 4A). Some overlap was observed between the two time-points in miRNA and lncRNA regulated transcripts in response to 4 Gy, 8 Gy and 10 Gy (Supplementary Fig. 4B,C). These data imply that expression levels of all three transcript types change in response to dose and time with major inter-transcript type differences too.

**Figure 2:**
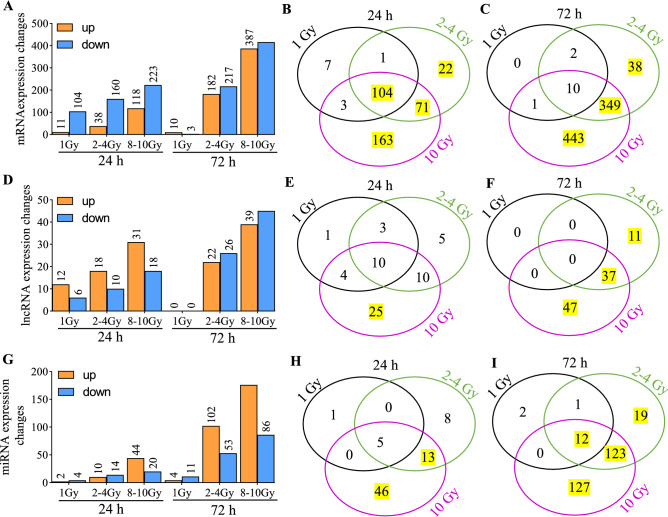
Summary of the differentially expressed RNAs when doses were grouped into three categories- 1 Gy, 2–4 Gy, and 8–10 Gy. Differentially expressed genes were identified as those fulfilling the criteria (FDR < 0.05; |FC|> 1.5). Genes which were induced are shown as orange bars and repressed genes are shown as blue bars in (**A,D,G**). (**A**) Differentially expressed mRNAs at 24 h and 72 h for 1 Gy, 2–4 Gy and 8–10 Gy, (**B**) Venn diagram showing overlaps across the upregulated mRNAs shown in (**A**), (**C**) Venn diagram showing overlaps across the downregulated mRNAs shown in (**A**). (**D**) Differentially expressed lncRNAs at 24 h and 72 h for 1 Gy, 2–4 Gy and 8–10 Gy, (**E**) Venn diagram showing overlaps across the upregulated mRNAs shown in (**D**), (**F**) Venn diagram showing overlaps across the downregulated mRNAs shown in (**D**). (**G**) Differentially expressed miRNAs at 24 h and 72 h for 1 Gy, 2–4 Gy and 8–10 Gy, (**H**) Venn diagram showing overlaps across the upregulated mRNAs shown in (**G**), (**I**) Venn diagram showing overlaps across the downregulated mRNAs shown in (**G**). All values greater than 10 are highlighted in yellow.

To add clinical correlation to the dose analysis, we grouped doses into three classes—low (1 Gy), where there might be mild symptoms but minimal risk of serious injury; medium (2 Gy, 4 Gy), where the acute radiation syndrome can occur and for which early intervention has life-saving potential; and high (8 Gy, 10 Gy), which would have a limited (8 Gy) to no (10 Gy) chance of survival. Biomarkers would be useful in triage and medical management, particularly important in a resource-limited setting. A dose dependent increase in regulated transcripts were observed (Fig. 2A, D, G). Across all classes, 104 mRNA transcripts were regulated at 24 h and 10 mRNA transcripts were regulated at 72 h (Fig. 2B, C). Ten miRNA transcripts were regulated in all groups at 24 h while none were common at the 72 h (Fig. 2E, F). Five and 12 lncRNA transcripts were commonly regulated across all classes at the 24 h and 72 h time-points respectively (Fig. 2H, I).

### Endothelial inherent radiation responsive markers and dose-differentiation markers

**Figure 3:**
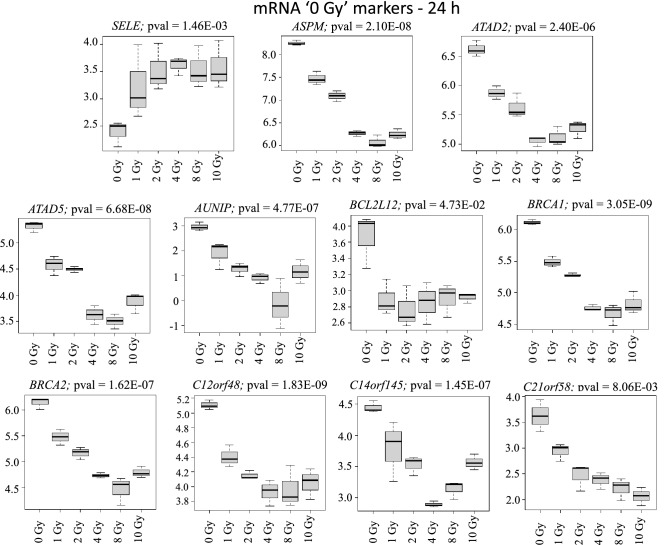
Radiation responsive mRNAs at 24 h for differentiating 0 Gy from all the doses classes. The 11 most significant mRNAs out of the 93 mRNAs at the intersection of all doses in supplementary Fig. 2A were selected as 0 Gy-24 h markers. Box-plots were generated in R. Y-axis shows the normalized read-counts and X-axis represents dose classes for 24 h time-point.

**Figure 4:**
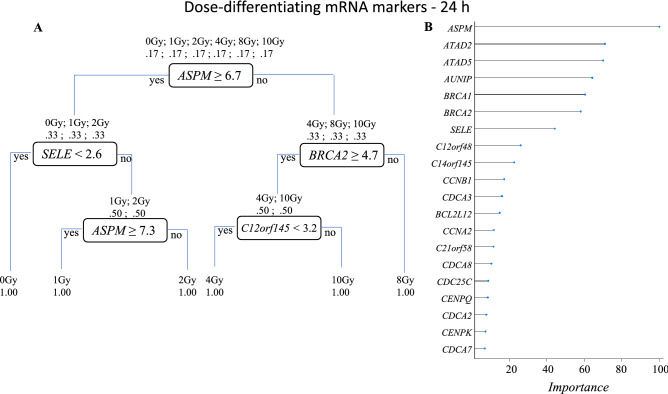
Dose-differentiation mRNA markers at 24 h. (**A**) Decision tree based on the RPART analysis performed in R for dose differentiation at 24 h based on the 93 mRNAs at the intersection of all doses in supplementary Fig. 2A. A combination of five mRNAs could differentiate all dose classes. Each node represents an mRNA intensity value based on which a decision is made, and classes are sorted. At the starting node there is a 17% chance of a sample falling into any of the six classes (0 Gy, 1 Gy, 2 Gy, 4 Gy, 8 Gy, 10 Gy). Decision at the first node divides the samples into two groups (0 Gy, 1 Gy, 2 Gy) and (4 Gy, 8 Gy, 10 Gy) at the second step. Two different mRNAs’ intensity values are used to divide the classes further. This process is repeated till all the classes are separated. (**B**) Plot shows relative importance of different mRNAs in dose-separation shown in (**A**).

**Figure 5:**
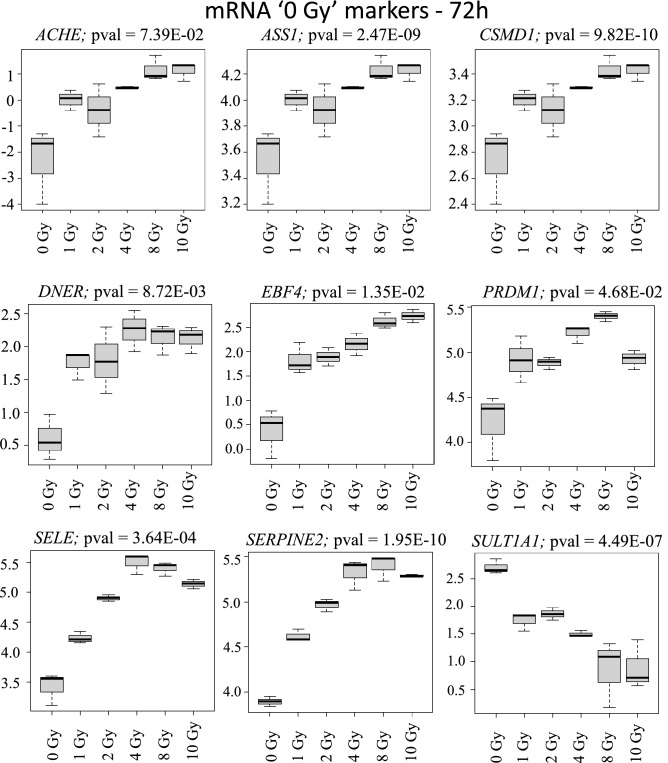
Radiation responsive mRNA at 72 h for differentiating 0 Gy from all the dosed classes. The nine most significant mRNAs out of the nine mRNAs at the intersection of all doses in supplementary Fig. 2B were selected as 0 Gy-72 h markers. Box-plots were generated in R. Y-axis shows the normalized read-counts and X-axis represents dose classes for 72 h time-point.

**Figure 6:**
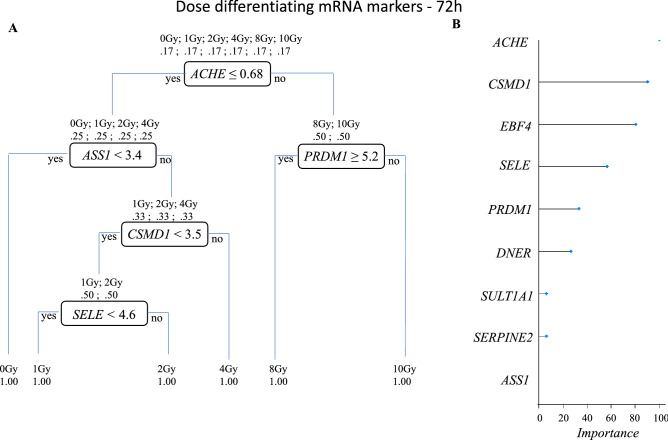
Dose-differentiation mRNA markers at 72 h. (**A**) Decision tree based on the RPART analysis performed in R for dose differentiation at 72 h based on the nine mRNAs at the intersection of all doses in supplementary Fig. 2B. A combination of five mRNAs could differentiate all dose classes. Each node represents an mRNA intensity value based on which a decision is made, and classes are sorted. At the starting node there is a 17% chance of a sample falling into any of the six classes (0 Gy, 1 Gy, 2 Gy, 4 Gy, 8 Gy, 10 Gy). Decision at the first node divides the samples into two groups (0 Gy, 1 Gy, 2 Gy, 4 Gy) and (8 Gy, 10 Gy) at the second step. Two different mRNAs’ intensity values are used to divide the classes further. This process is repeated till all the classes are separated. (**B**) Plot shows relative importance of different mRNAs in dose-separation shown in (**A**).

In mass radiological casualty scenarios, it will be crucial to identify the unexposed or concerned citizens from the exposed individuals for optimal resource management. Hence, identification of 0 Gy or *no-exposure vs. some exposure markers* is particularly useful. Focusing on the intersections across all doses for the three transcript types identified several 0 Gy markers and markers for dose-differentiation. Of the 93 commonly regulated mRNAs across all doses at 24 h time-point, we showed the 11 most-significantly regulated mRNAs (Fig. 3). These were *SELE*, *ASPM*, *ATAD*, *ATAD5*, *AUNIP*, *BCL2L12*, *BRCA1*, *BRCA2*, *C12orf48*, *C14orf145* and *C21orf58*. Because of the smaller replicate size and large numbers of dose-groups, a classification and prediction analysis was not performed. Dose-classification on these 93 mRNAs was performed using recursive partitioning for classification, regression tree (RPART) after five-fold CV. Four mRNAs (*ASPM*, *SELE*, *BRCA2* and *C14orf145*) could potentially classify the dose-classes successfully (Fig. 4). Similarly, nine mRNAs (*ACHE*, *ASS1*, *CSMD1*, *DNER*, *EBF4*, *PRDM1*, *SELE*, *SERPINE2* and *SULT1A1*) were most significantly differentially expressed in 0 Gy versus all the other dose points and could potentially serve as exposure/no-exposure markers at the 72 h (Fig. 5). Five of these mRNAs (*ACHE*, *ASS1*, *PRDM1*, *CSMD1* and *SELE*) classified all the dose classes in five steps successfully (Fig. 6). For validating RNAseq findings, we performed qRT-PCR on select genes from Figs. 4 and 6. The qRT-PCR results are shown in supplementary Fig. 5. 0 Gy markers were identified from miRNA and lncRNA data as well. Two miRNAs (miR-128-1-5p and miR-27a-5p) differentiated 0 Gy from all other doses at the 24 h time-point (Supplementary Fig. 6A). The ten 0 Gy differentiating miRNA markers at the 72 h time-point were miR-146a-5p, miR-181a-5p, miR-22-3p, miR-125b-1-3p, miR-222-5p, miR-27a-5p, miR-3529-3p, miR-7-5p, miR-7974-5p and miR-92a-1-5p (Supplementary Fig. 6B). RPART classification identified that three miRNAs (miR-146a-5p, miR-181a-5p and miR-3529-3p) could group dose classes in five steps (Supplementary Fig. 7). Five lncRNAs (CTD-216H16-4, RP11-E4.1, RP11-727A23.5, RP5-890E16.2, DLEU2 (Deleted in Lymphocytic Leukemia 2), HELLPAR (HELLP Associated lncRNA) and RP5-108D14.6) which were common across all doses in comparison to 0 Gy could also differentiate 0 Gy class from the rest of the data points at the 24 h time-point (Supplementary Fig. 8). No lncRNAs were found common to all doses at the 72 h time-point.

### Ingenuity pathway analysis and gene ontology for function identification based on altered mRNA transcript expressions

**Figure 7:**
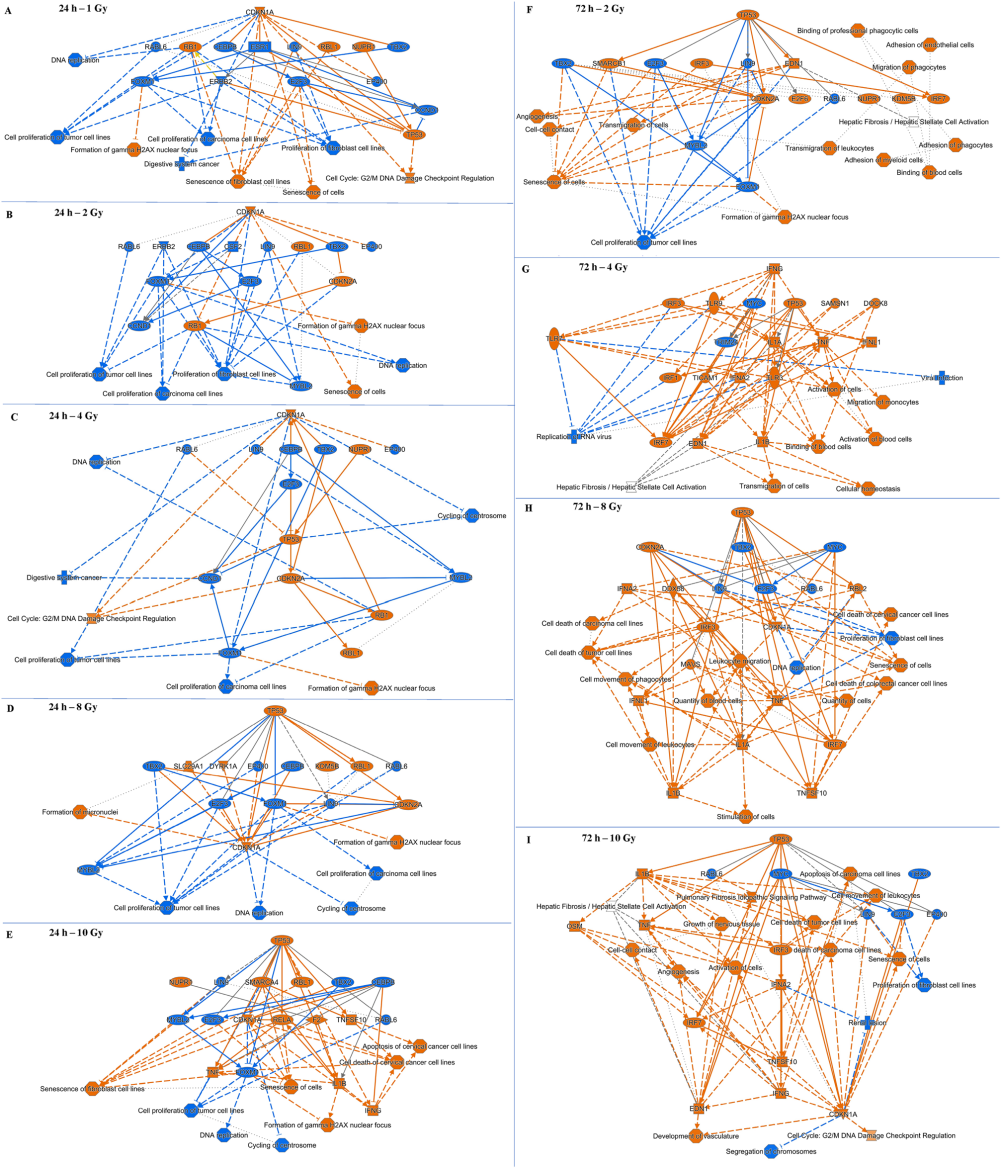
IPA generated graphical summaries for each dose-time-point combination. Core analysis was performed for each dose-time-point using differentially regulated mRNA lists. Graphical summaries include canonical pathways (Fisher’s exact test, p < 0.05) and upstream modulators (p < 0.05 and |z|= 2). Blue arrows and nodes indicate repressed activity; red arrows and nodes indicate induced activity; grey arrows suggest no definitive evidence on repression or activation. Solid arrows indicate direct interaction; dashed arrows indicate indirect interaction; dotted arrows indicate inferred and predicted interaction.

**Figure 8:**
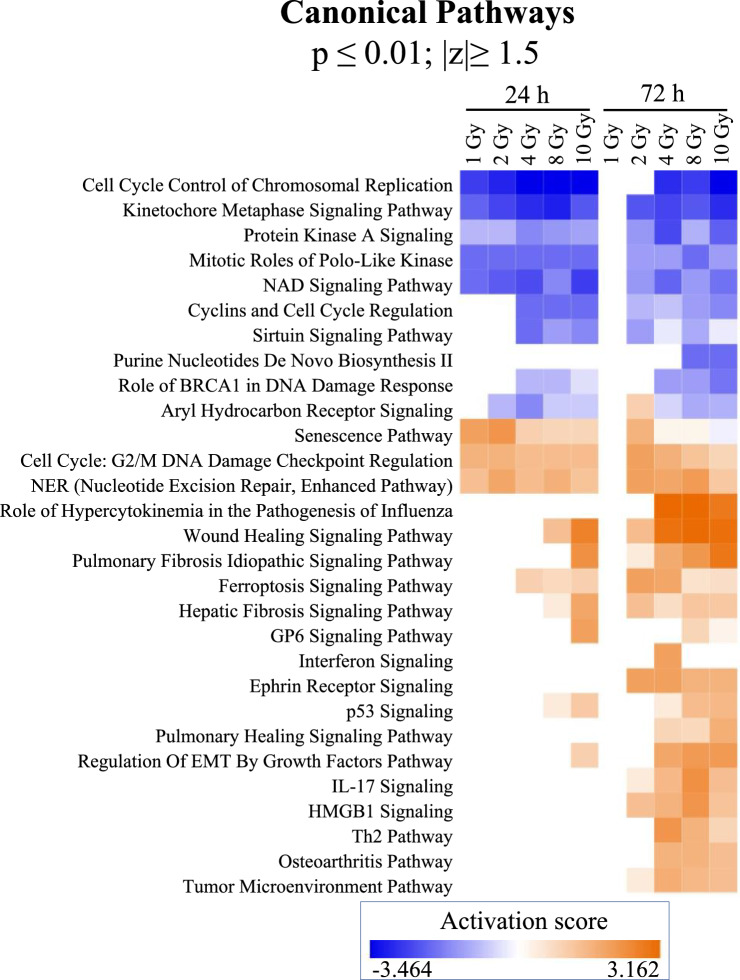
Comparison across canonical pathways identified in all dose-time-point combinations. Comparison was generated in IPA using the ‘comparison analysis’. Pathways passing the criteria of fisher’s test p value < 0.05 and |z-score|> 2 were included.

Ingenuity Pathway Analysis (IPA) core analysis was performed to identify pathways and key function terms in the mRNA data. An overview in the form of graphical summaries of the data for each dose-timepoint combination are shown in Fig. 8. At the 24 h time-point, for doses 1 Gy to 8 Gy, more downregulated nodes were observed suggesting a general depression of the gene expression (Fig. 7A–D). For 10 Gy exposure at 24 h though, major upregulated and activated nodes were observed (Fig. 7E). Since only 13 mRNAs were regulated in response to 1 Gy at 72 h, no significant alterations in any pathways or processes were detected by the IPA core analysis. But 2–10 Gy dose points at 72 h saw more upregulated and activated nodes compared to repressed and downregulated nodes (Fig. 7F–I). At the 24 h time-point, CDKN1A was the highest regulator for 1–4 Gy but P53 was the highest for 8 Gy and 10 Gy (Fig. 7A–E). At the 72 h time-point, P53 was the highest regulator at all doses except 4 Gy where IFNG was the highest regulator (Fig. 7F–I). Of the pathways, DNA damage response was predicted as activated and cell-cycle related signals were inhibited at the 24 h across all doses (Fig. 7A–E). At the 72 h, immune response was induced across all the doses (Fig. 7F–I).

Performing a comparison analysis across all dose-time-point combinations revealed several mitosis and cell-cycle related pathways such as kinetochore metaphase signaling, cell cycle control of chromosomal replication, mitotic roles of polo-like kinase, and protein kinase A signaling were down-regulated across all the doses at 24 h and 2 Gy- 4 Gy at the 72 h time point (Fig. 8). No pathways were regulated in response to 1 Gy at the 72 h time point. Stress induced pathways- senescence pathway, cell cycle G2/M DNA damage checkpoint regulation and NER (nucleotide excision repair) pathways were induced in all 24 h dose-points and 2–10 Gy—72 h dose-points. Several stress-activated and immune response pathways such as P53 signaling, IL-17 signaling, senescence pathway, ferroptosis pathway, HMGB1 signaling, Interferon signaling etc. were activated across 2–10 Gy at the 72 h and/or at 4–10 Gy at the 24 h. TP53 (Tumor protein p53), NUPR1 (Nuclear Protein 1, Transcriptional regulator), CDKN2A (Cyclin Dependent Kinase inhibitor 2A) and let-7 miRNA were identified as the top most activated upstream regulators based on the gene expression response while several growth factors and oncogenes such as MYC, ESR1 (Estrogen Receptor 1), YAP1 (Yes1 Associated Protein 1), E2F3 (E2F transcription factor 3), CCND1 (Cyclin D1), FOXM1 (Forkhead box protein M1), E2F1 (E2F transcription factor 1) and IL10 (Interleukin 10) were identified as the repressed upstream regulators across all doses at 24 h and 2–10 Gy at the 72 h (Supplementary Fig. 9A). Several upstream regulators were activated or repressed across 2–10 Gy at 72 h and only at higher doses of 8 Gy and/or 10 Gy at the 24 h (Supplementary Fig. 9B,C). Alfatoxin B1, calcitriol and l-asparaginase were the top upstream regulators (Supplementary Fig. 10). Similar to previous results several upstream regulators came up in higher doses (8 Gy and 10 Gy) at the 24 h time-point and across 2–10 Gy at the 72 h time-point (Supplementary Fig. 10). Disease and biofunction terms related to cell proliferation, DNA synthesis, M-phase, and several cancers were predicted as depressed while terms such as apoptosis, necrosis, death and senescence were predicted as activated (Supplementary Fig. 11).

For identifying Gene Ontology terms significant in our data, EnrichGO package in R was used. Helicase and kinase activity were prominent molecular function (MF) identified (Supplementary Fig. 12A). In biological processes (BP) we observed predominance of replication, division, chromosome segregation in downregulated gene lists across multiple dose groups (Supplementary Fig. 12B). Metabolism and immune process related terms such as ethanol metabolic process and leukocyte cell–cell adhesion were identified in the upregulated gene lists at 72 h (Supplementary Fig. 12B). These results bear high correlation to the IPA findings. The two methods validate each other. Chromosome, kinetochore, extracellular matrix, ER lumen, and lipid droplet were the cellular component (CC) terms significant in our data (Supplementary Fig. 12C).

### Correlation between differentially expressed miRNAs and mRNAs

We employed the IPA mRNA Target Filter to identify the differentially expressed mRNAs which were targets of the differentially expressed miRNAs and thus inversely regulated for each dose (Table 1). These targets were examined to study mechanisms and to act as an internal control for a biomarker. A down biomarker may be difficult to assay, but its correlative up biomarker enhances confidence for medical decision-making. No inversely correlated miRNA-mRNA pairs were found for 1 Gy and 2 Gy doses at the 24 h time-point. The general trend across all the data variables in the inversely paired miRNA-mRNAs was that the miRNA expression was induced while the target expression was repressed. Furthermore, while few miRNAs were consistently regulated across different doses, their inversely correlated target mRNAs were different. hsa-miR-1-3p was consistently induced and its mRNA targets *IQGAP3* (IQ Motif Containing GTPase Activating Protein 3) and *UHRF1* (Ubiquitin Like With PHD And Ring Finger Domains 1) were consistently repressed in response to 2–10 Gy at the 72 h time-point. Other targets like *POLA1* (DNA Polymerase A1), *NOTCH2* (Notch Receptor 2) and *BRI3BP* (BRI3 Binding Protein) were also downregulated in 4 Gy and higher doses. hsa-miR-942-5p was repressed at 4 Gy-24 h, 4 Gy-72 h and 8 Gy-72 h while its target mRNA *PLTP* (Phospholipid Transfer Protein) was induced at these dose-time-point combinations. hsa-miR-506-3p was induced in response to 8 Gy and 10 Gy at the 72 h time-point and several of its targets were downregulated. hsa-miR-100-3p was also repressed at 8 Gy and 10 Gy at 72 h and its targets *SOCS2* (Suppressor Of Cytokine Signaling 2) and *CYTL1* (Cytokine Like 1) were induced.

**Table 1:** Conversely regulated miRNAs and their mRNA targets identified using the miRNA Target Filter Analysis in IPA.

miRNA	miRNA log ratio	Gene target	Gene log ratio
**4 Gy—24 h**			
hsa-miR-942-5p	−0.594	PLTP	0.66
**8 Gy—24 h**			
hsa-miR-143-3p	*0.504*	PLK1	**−2.044**
hsa-miR-1246	*0.731*	DSCC1	**−2.08**
**10 Gy—24 h**			
hsa-let-7c-3p	*−0.561*	BTG1	**0.555**
**1 Gy—72 h**			
hsa-let-7c-1-3p	*−0.898*	SERPINE2	**0.729**
**2 Gy—72 h**			
hsa-let-7c-3p	*−0.57*	SERPINE2	**1.094**
hsa-miR-10b-5p	**0.643**	FAM111B	*−0.63*
hsa-miR-1-3p	**4.735**	IQGAP3	*−0.851*
hsa-miR-1-3p	**4.735**	UHRF1	*−0.814*
**4 Gy—72 h**			
hsa-miR-1908-5p	*−0.763*	VPS37D	**0.645**
hsa-miR-1908-5p	*−0.763*	HCN2	**0.768**
hsa-miR-1908-5p	*−0.763*	SEPTIN5	**0.787**
hsa-miR-1908-5p	*−0.763*	MDGA1	**1.172**
hsa-miR-1908-5p	*−0.763*	OLFM2	**2.2**
hsa-miR-1908-5p	*−0.763*	CYS1	**3.283**
hsa-miR-1908-5p	*−0.763*	GBP5	**4.183**
hsa-miR-1908-5p	*−0.763*	PRODH	**4.365**
hsa-miR-942-5p	*−0.591*	PLTP	**1.088**
hsa-miR-10b-5p	**0.584**	FAM111B	*−1.413*
hsa-miR-10b-5p	**0.584**	FIGN	*−0.599*
hsa-miR-1-3p	**5.176**	IQGAP3	*−1.72*
hsa-miR-1-3p	**5.176**	UHRF1	*−1.574*
hsa-miR-1-3p	**5.176**	ANP32B	*−1.136*
hsa-miR-1-3p	**5.176**	CKS1B	*−0.969*
hsa-miR-1-3p	**5.176**	AXL	*−0.84*
hsa-miR-1-3p	**5.176**	POLA1	*−0.815*
hsa-miR-1-3p	**5.176**	BRI3BP	*−0.731*
**8 Gy—72 h**			
hsa-miR-100-3p	*−1.525*	SOCS2	**0.766**
hsa-miR-100-3p	*−1.525*	CYTL1	**1**
hsa-miR-942-5p	*−0.786*	PLTP	**1.526**
hsa-miR-10a-5p	**0.587**	FAM111B	*−3.167*
hsa-miR-10a-5p	**0.587**	SNX22	*−2.093*
hsa-miR-10a-5p	**0.587**	SDC1	*−0.676*
hsa-miR-10a-5p	**0.587**	FIGN	*−0.659*
hsa-miR-10a-5p	**0.587**	TFRC	*−0.634*
hsa-miR-1197	**0.652**	PIR	*−0.996*
hsa-miR-506-3p	**3.194**	UHRF1	*−2.911*
hsa-miR-506-3p	**3.194**	CDCA7	*−2.78*
hsa-miR-506-3p	**3.194**	LMNB1	*−2.53*
hsa-miR-506-3p	**3.194**	LHX9	*−2.522*
hsa-miR-506-3p	**3.194**	DHCR24	*−1.542*
hsa-miR-506-3p	**3.194**	PRRX1	*−1.542*
hsa-miR-506-3p	**3.194**	TOM1L1	*−0.97*
hsa-miR-506-3p	**3.194**	STARD4	*−0.903*
hsa-miR-506-3p	**3.194**	PHF19	*−0.892*
hsa-miR-506-3p	**3.194**	CCDC86	*−0.813*
hsa-miR-506-3p	**3.194**	NAA15	*−0.757*
hsa-miR-506-3p	**3.194**	PAQR8	*−0.589*
hsa-miR-506-3p	**3.194**	DNAJC25-GNG10	*−0.586*
hsa-miR-1-3p	**4.135**	IQGAP3	*−3.584*
hsa-miR-1-3p	**4.135**	UHRF1	*−2.911*
hsa-miR-1-3p	**4.135**	CKS1B	*−1.565*
hsa-miR-1-3p	**4.135**	AXL	*−1.235*
hsa-miR-1-3p	**4.135**	BRl3BP	*−1.041*
hsa-miR-1-3p	**4.135**	POLA1	*−1.006*
hsa-miR-1-3p	**4.135**	ANP32B	*−0.856*
hsa-miR-1-3p	**4.135**	CCSAP	*−0.813*
hsa-miR-1-3p	**4.135**	NOTCH2	*−0.728*
hsa-miR-1-3p	**4.135**	TDP1	*−0.675*
hsa-miR-1-3p	**4.135**	C2orf69	*−0.585*
**10 Gy—72 h**			
hsa-miR-100-3p	*−1.321*	SOCS2	**0.807**
hsa-miR-100-3p	*−1.321*	CYTL1	**1.259**
hsa-miR-942-5p	*−0.697*	CREG1	**0.662**
hsa-miR-942-5p	*−0.697*	IL1RAPL1	**0.938**
hsa-miR-942-5p	*−0.697*	PLTP	**1.541**
hsa-let-7f-5p	**0.608**	RRM2	*−4.639*
hsa-let-7f-5p	**0.608**	UHRF1	*−3.227*
hsa-let-7f-5p	**0.608**	PRIM1	*−1.616*
hsa-let-7f-5p	**0.608**	DOCK5	*−1.238*
hsa-let-7f-5p	**0.608**	HMGA1	*−1.072*
hsa-let-7f-5p	**0.608**	FIGN	*−1.021*
hsa-let-7f-5p	**0.608**	IGF2BP2	*−0.848*
hsa-let-7f-5p	**0.608**	STARD4	*−0.807*
hsa-let-7f-5p	**0.608**	MYC	*−0.734*
hsa-let-7f-5p	**0.608**	HMOX1	*−0.721*
hsa-let-7f-5p	**0.608**	MARS2	*−0.612*
hsa-miR-10b-5p	**0.905**	FAM111B	*−3.82*
hsa-miR-10b-5p	**0.905**	FIGN	*−1.021*
hsa-miR-10b-5p	**0.905**	SDC1	*−0.58*
hsa-miR-122-5p	**2.275**	SLC25A34	*−1.422*
hsa-miR-122-5p	**2.275**	SLC7A1	*−0.861*
hsa-miR-122-5p	**2.275**	FAM117B	*−0.582*
hsa-miR-122b-3p	**2.275**	NEK7	*−0.683*
hsa-miR-122b-3p	**2.275**	NAP1L5	*−0.583*
hsa-miR-506-3p	**3.51**	UHRF1	*−3.227*
hsa-miR-506-3p	**3.51**	LHX9	*−2.869*
hsa-miR-506-3p	**3.51**	CDCA7	*−2.84*
hsa-miR-506-3p	**3.51**	LMNB1	*−2.58*
hsa-miR-506-3p	**3.51**	PRRX1	*−1.676*
hsa-miR-506-3p	**3.51**	DHCR24	*−1.575*
hsa-miR-506-3p	**3.51**	CCDC86	*−1.006*
hsa-miR-506-3p	**3.51**	TOM1L1	*−0.992*
hsa-miR-506-3p	**3.51**	QSER1	*−0.901*
hsa-miR-506-3p	**3.51**	STARD4	*−0.807*
hsa-miR-506-3p	**3.51**	PHF19	*−0.795*
hsa-miR-506-3p	**3.51**	NAA15	*−0.698*
hsa-miR-506-3p	**3.51**	ELK3	*−0.613*
hsa-miR-506-3p	**3.51**	SLC7A2	*−0.598*
hsa-miR-506-3p	**3.51**	PAQR8	*−0.597*
hsa-miR-506-3p	**3.51**	DNAJC25-GNG10	*−0.595*
hsa-miR-506-3p	**3.51**	OAF	*−0.577*
hsa-miR-1-3p	**4.712**	IQGAP3	*−4.008*
hsa-miR-1-3p	**4.712**	UHRF1	*−3.227*
hsa-miR-1-3p	**4.712**	CKS1B	*−1.606*
hsa-miR-1-3p	**4.712**	AXL	*−1.298*
hsa-miR-1-3p	**4.712**	ANP32B	*−1.245*
hsa-miR-1-3p	**4.712**	BRI3BP	*−1.054*
hsa-miR-1-3p	**4.712**	NOTCH2	*−1.006*
hsa-miR-1-3p	**4.712**	POLA1	*−0.976*
hsa-miR-1-3p	**4.712**	NFATC2	*−0.714*
hsa-miR-1-3p	**4.712**	SLC25A22	*−0.671*
hsa-miR-1-3p	**4.712**	G6PD	*−0.652*
hsa-miR-1-3p	**4.712**	CCSAP	*−0.601*

For each dose-time-point combination inversely regulated miRNA-mRNA target pairs were identified in miRNA Target Filter analysis based on experimentally validated miRNA-mRNA interactions from mirTarBase and high-confidence predicted interactions from TargetScan. Bold indicate fold change ≥ 1.5 while italics indicate fold change ≤ 1.5. Only pairs with inverse fold change values were included.

## Discussion

Radiation-induced vascular or endothelial damage underlies normal tissue injury. For developing biomarkers for triage and treatment of individuals following whole body radiation exposure (i.e., radiation biodosimetry), including potential mitigators specifically for vascular injury, it is necessary to identify damage in a timely manner. Toward that end we performed a whole transcriptome RNA sequencing study to identify miRNAs, mRNAs and lncRNAs expressed in a radiation dose and time dependent manner in cultured primary human aortic endothelial cells. The dose range chosen for the study is based on whole body radiation responses: 1 Gy likely produces symptoms but little acute risk (it does increase lifetime risk for developing cancer); 2–4 Gy are where mitigators of hematological toxicity (the acute radiation syndrome- ARS) are needed to avert the risk of bone marrow toxicity related infection and death; 8–10 Gy will likely result in death in the first few weeks-to- months and certainly months to years later from the delayed effect of acute radiation exposure (DEARE) with fibrosis and organ failure^39^. and > 10 Gy is almost invariably fatal within days^40^.

A critical distinction for triage is being able to define a population that does not need medical care for radiation exposure. We identified several mRNAs (*SELE*, *ASPM*, *ATAD*, *ATAD5*, *AUNIP*, *BCL2L12*, *BRCA1*, *BRCA2*, *C12orf48*, *C14orf145*, *C21orf58, ACHE, ASS1, CSMD1, DNER, EBF4, PRDM1, SERPINE2 and SULT1A1*), miRNAs (miR-128-1-5p, miR-146a-5p, miR-181a-5p, miR-22-3p, miR-125b-1-3p, miR-222-5p, miR-27a-5p, miR-3529-3p, miR-7-5p, miR-7974-5p and miR-92a-1-5p) and lncRNAs (CTD-216H16-4, RP11-E4.1, RP11-727A23.5, RP5-890E16.2, DLEU2, HELLPAR and RP5-108D14.6) which can differentiate unirradiated from dosed samples. Of these, *HELLPAR* has been shown to play a role in the manifestation of HELLP syndrome, an endothelial cell related syndrome that occurs during pregnancy^41^. Interestingly, none of these lncRNA have previously been shown to be regulated in a radiation-dependent manner. *SELE*, *ASPM*, *ATAD*, *ATAD5*, *AUNIP*, *CSMD1*, *DNER* and *EBF4* are also novel targets of radiation identified in this study.

The IPA analyses show that at the shorter time-point of 24 h, a general repressive theme emerged in response to 1–8 Gy even though the most important nodes were identified as activated (CDKN1A—1–4 Gy; P53—8 Gy). In response to 10 Gy however, IPA analysis revealed general activation orchestrated by P53. In contrast, three days post-radiation, the general theme across all doses was one of activation orchestrated by P53 at all doses except 4 Gy (IFNG was highest node at 4 Gy). We would interpret these data as follows. Immediate response of the endothelial cells from low (1 Gy) to medium–high doses (8 Gy) is to shut-down cellular machinery. But by the 72 h time-point, cells have set into motion pathways and axes of repair and regeneration in order to rectify the damage induced. At the highest dose (10 Gy), however, cells are responding at the 24 h in a similar way that they respond at the 72 h for even the lower doses. This suggests speedier orchestration of repair pathways when damage sustained is very high. Delving into the identity of the node points suggests that primarily, cells have stalled DNA replication and all cell-division related signals at the 24 h time-point for 1–8 Gy. But in response to 10 Gy, cells in addition have also activated apoptosis and immune related signals (TNF, IL1B and IFNG). Immune signals were identified at the 72 h for all doses as well. At the 2 Gy dose, endothelial cells were projected to secrete signals which potentially could influence processes such as adhesion and migration of different blood cell types. For 4–10 Gy doses, these potential effects seem to have strengthened by secretion of more diverse interleukins and other cytokines. Angiogenesis and senescence were other processes which were predicted by IPA at the 72 h time-point. These various data together might imply the following. One day after radiation, endothelial cells were involved in immediate resolution of direct radiation damage by stalling cell-cycle pathways. But by the third day, cells were sending out signals for immune cells to further repair the damage. Angiogenesis signals were generated to repair whatever gaps were created in the endothelial monolayer. Senescent signals were another way endothelial cells reacted to endure and survive the radiation damage^31^. Similar pathway differences have been observed by other groups in endothelial cells in response to radiation^27,30,31^.

Regarding mechanism of injury, cumulative observation of the pathways disrupted over time indicated that radiation exposure affected apoptotic, mitotic and senescent pathways across all dose-timepoint combinations. Immune-related signals were observed at higher doses at 24 h and across all doses greater than 2 Gy at the 72 h. While induction of apoptotic and necrotic pathways was expected, deregulation of mitosis-related pathways was not. Endothelial cells achieve growth arrest on reaching confluence. It appears that radiation exposure activates proliferation in endothelial cells, which has previously been observed by several groups too^42^. Endothelial cells release increased amounts of growth factors such as PDGF in response to radiation^42^. Endothelial and smooth muscle cell proliferation after radiation has been linked to vascular occlusion and onset of fibrosis^42^. We speculate that radiation damage caused cell-death creates gaps across the endothelial cell monolayer and cells activate their cell-cycle pathways to repair those gaps. It is possible that the inability to divide, owing to excessive DNA damage even though cell-cycle pathways were activated, led to the observed induction of senescence and fibrosis pathways^43^.

To summarize the important observation of this study for mass casualty radiation biodosimetry is a time- and dose-dependent activation which is instructive for designing triage decisions and planning immediate therapies. These findings could help establish a point-of-care assay for delineating ‘0 Gy’ or the unexposed populations, who need no further assessment of radiation injury, besides differentiating doses based on a decision tree approach.

Future studies would investigate if these markers could inform development of a biodosimetry assay for differentiating radiation-exposed from those concerned citizens/not-exposed. Pathway analysis presented an interesting picture where cell-cycle stalling processes happened one day post-radiation and immune response orchestration was predicted three days post-radiation. This suggests that prudent application of radiation mitigation therapies would require understanding both dose and time-point effects. We plan to expand these investigations to longer times after radiation in our future studies. To learn if these changes are reflected in secretions of endothelial cells, we are characterizing extracellular vesicles originating from these cells. While speculative, the dose-time-effect information might have utility in the development of biomarkers for clinical radiotherapy to understand both how and when in the course of treatment endothelial cells respond/adapt to the radiation treatment.

## Materials and methods

### Cell culture and radiation

Primary human aortic endothelial cells were purchased from Cell Applications, Inc (California, US). All experiments were performed within five-six passages. Cells were grown and maintained in human EC Growth Medium (# 211-500, Cell Applications, Inc, US). Subculturing reagent kits (# 090K, Cell Applications Inc., US) comprising of Hank’s buffered saline solution (HBSS), Trypsin/EDTA, and trypsin neutralizing solution were used for expanding the cells. For irradiation, cells were plated in 60 mm dishes and allowed to reach confluence. Media was changed and cells were irradiated in an X-ray irradiator for various doses (0 Gy, 1 Gy, 2 Gy, 4 Gy, 8 Gy and 10 Gy). Irradiation was delivered at room temperature using 320 kV X-rays at a dose rate of 2.3 Gy/min from the PANTAK high frequency X-ray generator (Precision E-ray Inc., N. Bedford, CT). The irradiator is periodically serviced, calibrated and maintained (R3 X-Ray, LLC, Hudson, FL). No additional filters were used to modify the x-ray beams. Cells were incubated post-exposure for 24 and 72 h. Cell-lysates were prepared using Trizol (Qiazol, Qiagen) at the end of the time-points and stored in -80C until further processing. All experiments were performed in triplicates (n = 3; N = 36) at both time-points.

### RNA isolation

RNA was isolated using the miRNeasy kits (#217004, Qiagen) following the manufacturer’s protocol. Briefly, cells were lysed in 700 µl Qiazol Lysis Reagent. 140 µl chloroform was added and tubes were vigorously shaken. Tubes were centrifuged at 12,000*g* for 15 min at 4 °C for phase separation. The upper aqueous phase was transferred into a fresh tube and 1.5 volumes of 100% ethanol was added to precipitate out the RNA. The solution was loaded onto the RNeasy Mini columns and centrifuged at 12,000*g* for 30 s. RNA was left bound to the columns while the liquid phase passed through. RNA was washed with buffers RWT and RPE to remove any bound salts and impurities. The purified RNA was eluted by loading 20–30 µl of RNase-free water onto the column. RNA concentrations were quantified using Nanodrop spectrometer and RNA quality was assessed using Bioanalyzer.

### lncRNA inclusive library preparation and sequencing

lncRNA sequencing was performed by Novogene (Novogene, Sacramento, CA) using the TruSeq Stranded Total RNA Library Prep Kit (Illumina, US; catalogue # 20020597) with Illumina Ribo-Zero Plus rRNA Depletion kit (Illumina, US; catalogue # 20037135) following manufacturer’s instructions. Sequencing runs were performed on Novaseq 6000 S4 system (Illumina, US). Briefly, Poly-A tailed RNA was purified from total RNA using poly-T oligo attached magnetic beads. The purified RNA was fragmented and reverse-transcribed into first strand cDNA using random primers. Second strand cDNA synthesis was performed using dUTP to preserve strand specific information. 3’-ends were repaired with adenylation. Adapters were ligated for strand selection and sequencing priming. PCR amplification was followed by purification. The quality of the amplified library was assessed on Bioanalyzer, and libraries were quantified on Qubit Fluorometer (Themo Fisher Scientific, US). Sequencing reactions were performed to generated 150 bp paired-end reads.

### lncRNA sequencing data quality assessment and analysis

The original image data file was transformed into sequence reads (raw reads) by CASAVA base recognition software. Raw reads were stored as FASTQ files containing read sequences and corresponding base quality. All samples had Q30 Phred score > 93% and Q20 Phred score > 97%. Raw reads were filtered to remove adaptor containing reads, reads containing > 10% undetermined bases and reads with quality score of less than five for 50% bases. The clean reads were aligned to the human reference genome hg38 by HISAT2 v2.0.4. Greater than 75% of clean reads mapped to the genome in all samples. HTSeq was used to identify distribution of the mapped reads to known RNAs. rMATS v3.2.5 was used to distribute reads to alternatively spliced transcripts. StringTie v1.3.1 software was used to assemble and quantify the expression of isoforms keeping the isoform set as small as possible. Fragments per kilobase of transcript sequence per million bases sequenced (FPKM) levels were calculated for differential gene expression analysis. DEseq was used to identify transcripts differentially expressed in each condition compared to 0 Gy control samples satisfying an adjusted p-value < 0.05 and fold change ≥ 1.5.

### lncRNA filtering

Transcripts with potential lncRNA functions were filtered. Low confidence single exon transcripts were discarded. Transcripts longer than 200 bp were selected. Annotations were added for known lncRNA transcripts in Cuffcompare v2.1.1 and transcripts with FPKM < 0.5 were removed. Finally, coding potential filter was applied, and 2186 transcripts were identified as lncRNAs by CPC cpc-0.9-r2, CNCI v2, Pfam Analysis v1.3 and PhyloCSF v2012028 softwares.

### Small RNA seq library preparation

The small RNA libraries were prepared using the Qiaseq miRNA Library Preparation Kit (Qiagen, Hilden, Germany; catalogue no. 331505) following manufacturer’s instructions. Briefly, a universal preadenylated DNA adapter is added to the 3’ ends and an RNA adapter is ligated to the 5’ end. cDNA was synthesized using 3’-adapter as the primer. cDNA was subsequently cleaned up using magnetic QIAseq beads (provided with the library prep kit) and libraries were amplified and different 6-nt indices were included for each sample to enable sample identification after multiplexing. Library sizes were assessed using Bioanalyzer and average library sizes were around 180 bp. Samples were pooled and sequenced on Illumina Hiseq X sequencer generating 10 million single end reads per sample.

### Small RNAseq data quality assessment and analysis

Original image data files from high throughput sequencing were translated into sequence reads by CASAVA base calling. These raw reads were stored as FASTQ files containing the sequences and corresponding base quality. The Q20 and Q30 Phred scores for all samples were above 98%. Reads with low quality, containing adaptors, and containing > 10% uncertain bases were removed. Length distribution of data revealed highest concentration of RNAs between 20 and 24 nt length for all samples. The filtered reads were mapped to the human genome hg38 using Bowtie. Reads mapping to rRNA, tRNA, snRNA and snoRNA were removed. The remaining mapped reads were compared with specific sequences in miRbase to obtain information on well annotated miRNAs. sRNA reads aligning to repetitive sequence of the genome were also removed. Reads aligning to the exons and introns of mRNAs were also removed. Novel miRNA sequences were predicted using miREvo and mirdeep2 algorithms. The expressions of known and uniquely identified miRNAs were normalized by TPM (transcripts per million) for each sample as per the formula raw count*1,000,000/library size. Library size being the total sample miRNA counts. Differentially expressed miRNAs were identified in DESeq2 satisfying an adjusted p-value < 0.05 between pair-wise comparison of each dose with 0 Gy control samples.

### Statistical analysis for 0 Gy markers and dose-differentiating markers

Differential expression analysis was performed using glmLRT of edgeR package in R. Input for edgeR package is the count data that will be sequentially processed for gene length correction followed by linear model of the negative binomial family. The most significantly regulated genes across all doses at either 24 h or 72 h were selected by applying linear regression model with FDR ≤ 0.05 and log2 |FC|> 1.5. using tree model in rpart package in R. Importance for the biomarker signatures was measured after threefold cross validation by Bagging (Bootstrap Aggregating) algorithm to improve model accuracy in regression and classification problems. Bagging is one of the ensemble learning methods where multiple models made from separated subsets of train data, and constructing a final aggregated and more accurate model by bagging algorithm. Importance was measured as an index of the reduction in the loss of function (e.g., mean squared error) attributed to each variable at each split which was tabulated as the sum.

### Data availability

All the sequencing data and methodology details have been submitted to GEO under the accession number GSE202121. It includes sub-series GSE202119 and GSE202120 for small RNA seq and lncRNA seq data.

### Quantitative RT-PCR

qRT-PCR was performed for a select set of genes on an independent set of samples radiated with either 0 Gy, 1 Gy, 2 Gy, 4 Gy, 8 Gy or 10 Gy for 24 h and 72 h (n = 4; N = 48). One µg of RNA was reverse transcribed using the RT2 First Strand kit (Qiagen, US). 10 ng cDNA was used in each reaction for PCR reactions using RT2 SYBR Green qPCR master mix (Qiagen, US). Gene primers were purchased from Qiagen and are listed here: *CSMD1* (PPH19714A), *PRDM1* (PPH18652C), *EBF4* (PPH15569B), *DNER* (PPH14738A), *ASS1* (PPH13206E), *ACHE* (PPH12939A), *ASPM* (PPH1519B), *SERPINE2* (PPH08354A), *SULT1A1* (PPH01565B), *SELE* (PPH00683F), *BRCA2* (PPH00321F), *ATAD5* (PPH17239A), *ATAD2* (PPH16398A), *BCL2L12* (PPH16195F), *AUNIP* (PPH12740A), and *BRCA1* (PPH00322F). PCR reactions were performed in ABI QuantStudio. *GAPDH* (Qiagen, PPH00150F) was used as the normalizing gene. Fold change values were calculated as follows:

$$\begin{gathered} {\text{dCT}}_{{{\text{gene}}\_{\text{sampleA}}}} = {\text{ CT}}_{{{\text{gene}}\_{\text{sampleA}}}} {-}{\text{ CT}}_{{{\text{GAPDH}}\_{\text{sampleA}}}} \hfill \\ {\text{ddCT}}_{{{\text{gene}}\_{\text{sampleA}}}} = {\text{ dCT}}_{{{\text{gene}}\_{\text{sampleA}}}} {-}{\text{ dCT}}_{{{\text{gene}}\_{\text{control sample}}}} \hfill \\ {\text{Fold change}}_{{{\text{gene}}\_{\text{sampleA}}}} = { 2}^{{ - {\text{ddCT}}}}_{{{\text{gene}}\_{\text{sampleA}}}} \hfill \\ \end{gathered}$$

where CT is the threshold cycle number. Statistical significance was calculated by performing unpaired students’ two-tailed t-test between control and each radiation sample.

### Ingenuity pathway analysis

Pathway analysis was performed in Ingenuity Pathway Analysis (IPA) (Qiagen, US). Differentially expressed mRNA and miRNA lists were uploaded and core analyses were performed on each pair-wise comparison. Graphical summaries were generated which represent the most valuable gist of the data since pathways included in the summary must pass a fisher’s exact test (P < 0.05) and all upstream regulators must pass an additional cut-off of absolute z-score at two. Machine learning algorithms then help decide which nodes to prioritize, which connections have been established in the literature and which connections could be inferred anew. Comparisons of the most important signaling pathways and regulators in the form of graphical summaries revealed interesting patterns and differences along both dose and time axes. Comparison analyses were performed across all mRNA core analyses and miRNA core analyses outputs. miRNA Target Filter (IPA, Qiagen) analysis was used to find targets of differentially expressed miRNAs from the differentially expressed mRNA lists for each pair-wise comparison.

## Supplementary Information

Supplementary Figure 1.

Supplementary Figure 2.

Supplementary Figure 3.

Supplementary Figure 4.

Supplementary Figure 5.

Supplementary Figure 6.

Supplementary Figure 7.

Supplementary Figure 8.

Supplementary Figure 9.

Supplementary Figure 10.

Supplementary Figure 11.

Supplementary Figure 12.

Supplementary Legends.
