# Supplementary material for: Profiling mRNA, miRNA and lncRNA expression changes in endothelial cells in response to increasing doses of ionizing radiation
Source: Sci Rep. 2022 Nov 19;12:19941. doi: 10.1038/s41598-022-24051-6 (PMC9675751; doi:10.1038/s41598-022-24051-6)
Supplement: Supplementary file 1 — Supplementary Figure 1. [file 41598_2022_24051_MOESM1_ESM.pdf]

A **mRNA**

Supplementary Figure 1: Principal component analysis (PCA) plots were generated in R using gglot2 on the normalized read counts for A) mRNA, B) lncRNA, and C) miRNA data.

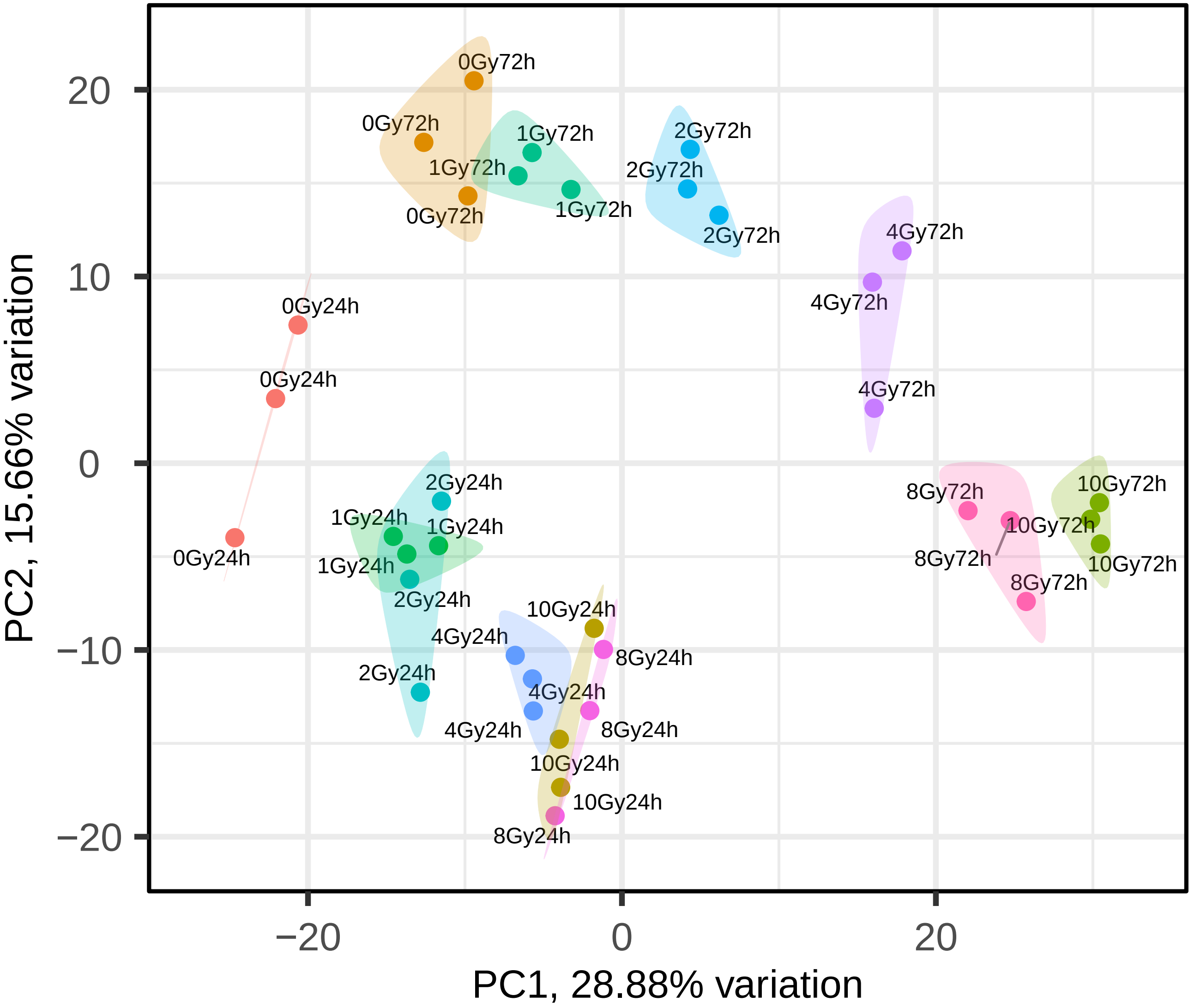

B

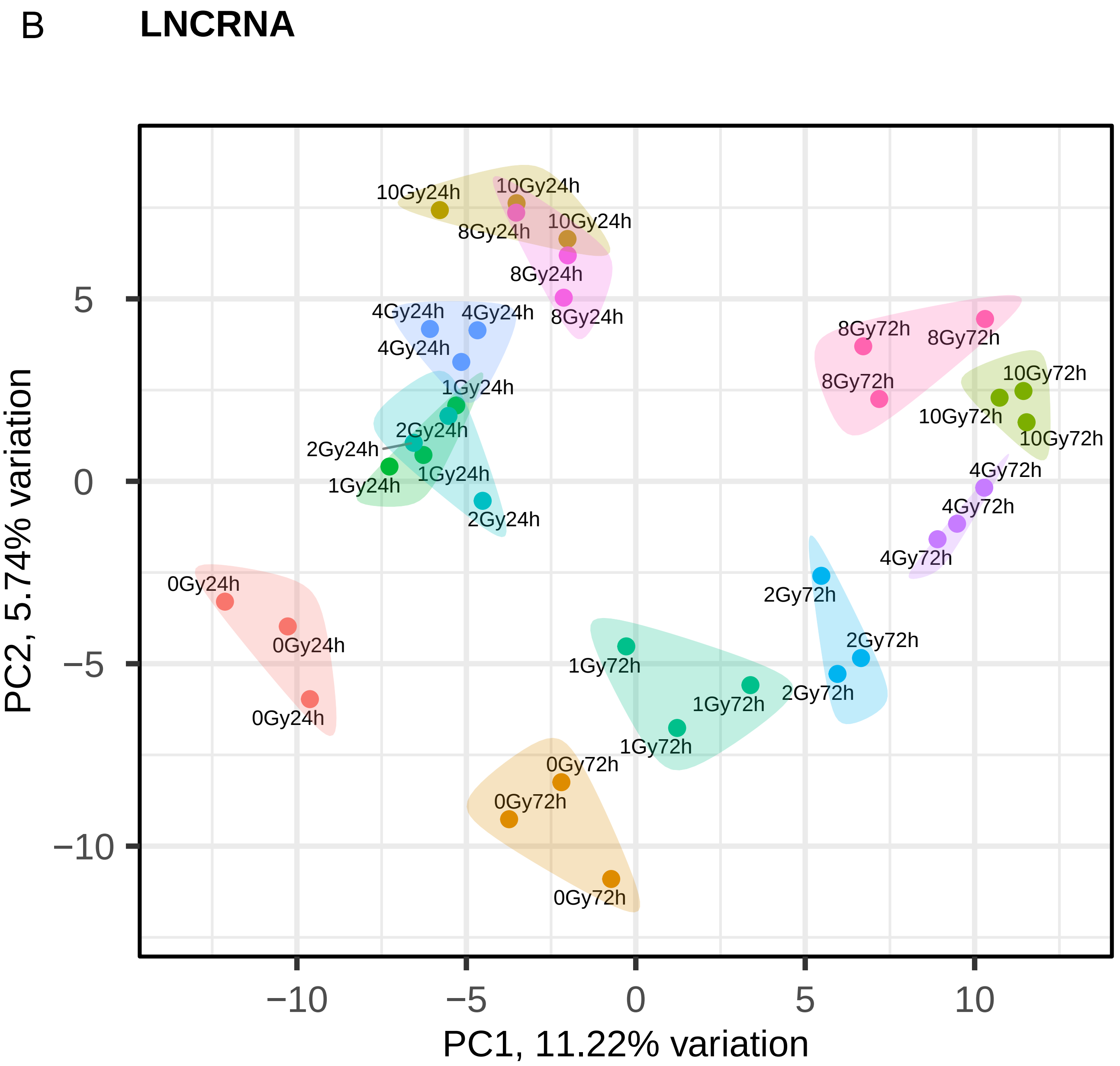

LNCRNA
PC1, 11.22% variation
PC2, 5.74% variation
−10
−5
0
5
10
10Gy24h
10Gy24h
10Gy24h
8Gy24h
8Gy24h
8Gy24h
4Gy24h
4Gy24h
4Gy24h
1Gy24h
1Gy24h
1Gy24h
2Gy24h
2Gy24h
2Gy24h
0Gy24h
0Gy24h
0Gy24h
8Gy72h
8Gy72h
8Gy72h
10Gy72h
10Gy72h
10Gy72h
4Gy72h
4Gy72h
4Gy72h
2Gy72h
2Gy72h
2Gy72h
1Gy72h
1Gy72h
1Gy72h
0Gy72h
0Gy72h
0Gy72h

C **miRNA**

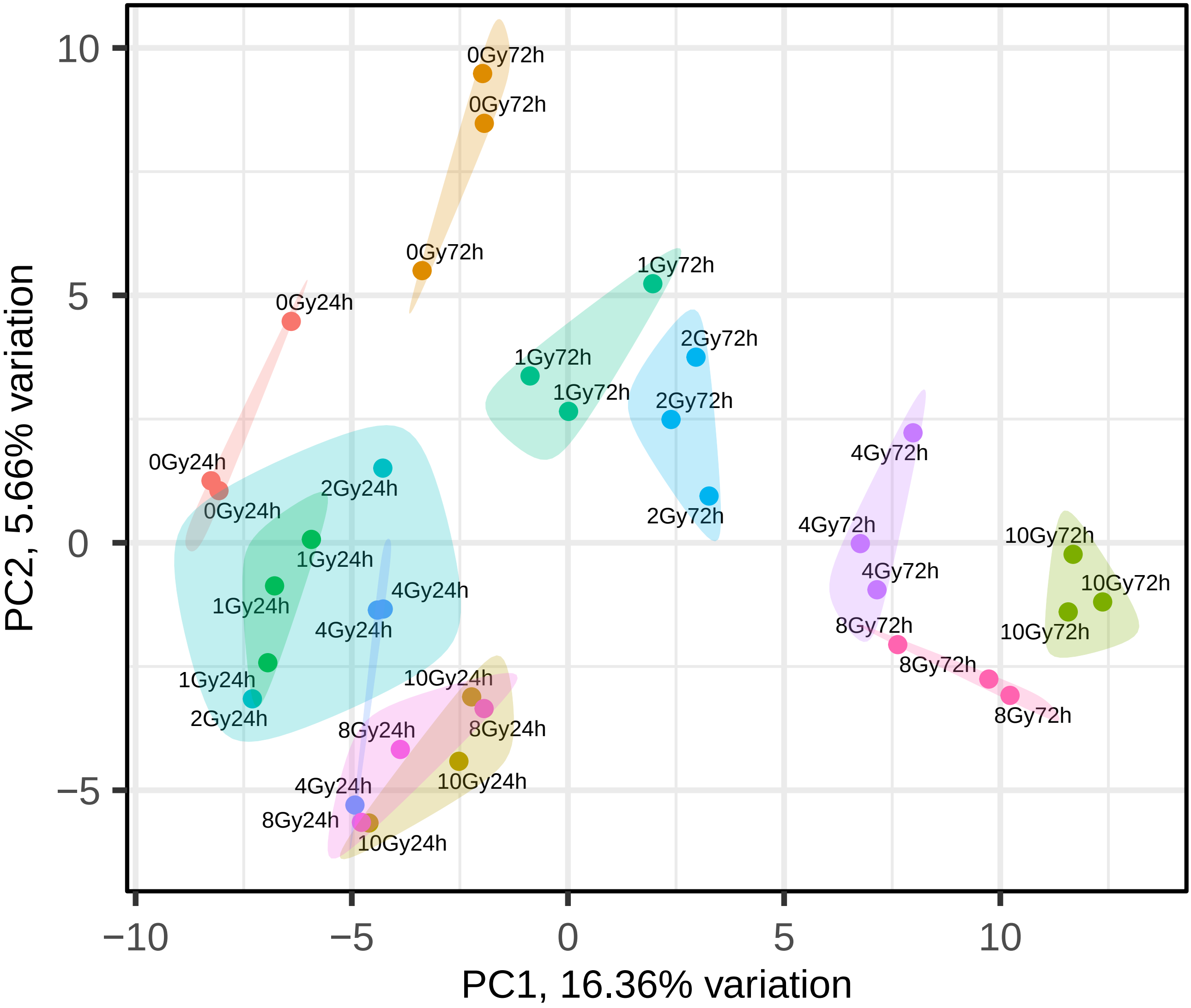

PC1, 16.36% variation
PC2, 5.66% variation
−10
−5
0
5
10
0Gy72h
0Gy72h
0Gy72h
0Gy24h
0Gy24h
0Gy24h
1Gy72h
1Gy72h
1Gy72h
2Gy72h
2Gy72h
2Gy72h
2Gy24h
1Gy24h
1Gy24h
1Gy24h
2Gy24h
4Gy24h
4Gy24h
4Gy24h
4Gy72h
4Gy72h
4Gy72h
10Gy72h
10Gy72h
10Gy72h
8Gy72h
8Gy72h
8Gy72h
10Gy24h
8Gy24h
8Gy24h
10Gy24h
8Gy24h
10Gy24h
