# Supplementary material for: Profiling mRNA, miRNA and lncRNA expression changes in endothelial cells in response to increasing doses of ionizing radiation
Source: Sci Rep. 2022 Nov 19;12:19941. doi: 10.1038/s41598-022-24051-6 (PMC9675751; doi:10.1038/s41598-022-24051-6)
Supplement: Supplementary file 2 — Supplementary Figure 2. [file 41598_2022_24051_MOESM2_ESM.pptx]

## Slide 1
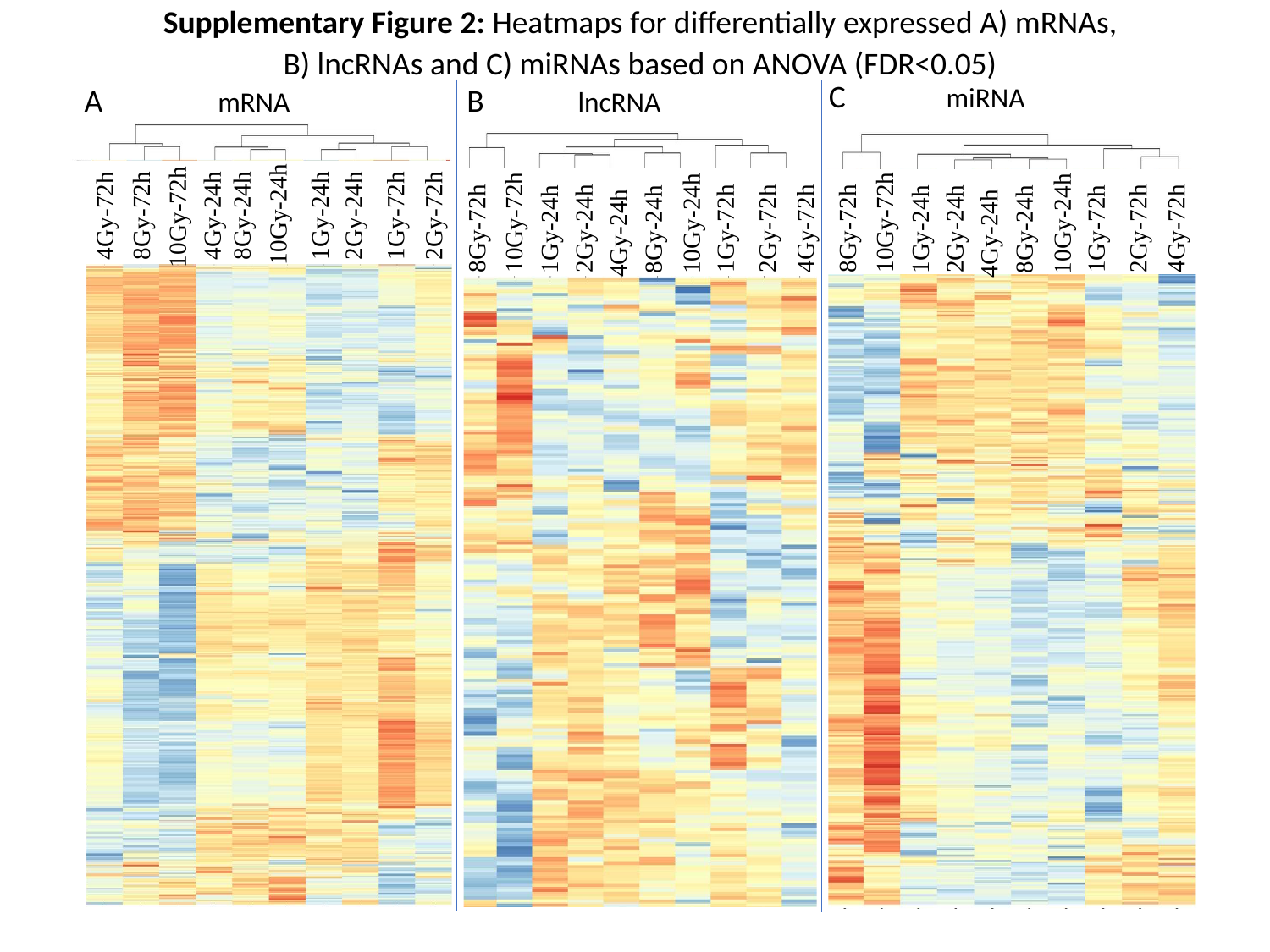

Supplementary Figure 2: Heatmaps for differentially expressed A) mRNAs, B) lncRNAs and C) miRNAs based on ANOVA (FDR<0.05)
C miRNA
A mRNA
B lncRNA
4Gy-72h
8Gy-72h
10Gy-72h
4Gy-24h
8Gy-24h
10Gy-24h
1Gy-24h
2Gy-24h
1Gy-72h
2Gy-72h
1Gy-24h
10Gy-72h
8Gy-24h
10Gy-24h
8Gy-72h
2Gy-24h
1Gy-72h
2Gy-72h
4Gy-72h
4Gy-24h
1Gy-24h
10Gy-72h
8Gy-24h
10Gy-24h
8Gy-72h
2Gy-24h
1Gy-72h
2Gy-72h
4Gy-72h
4Gy-24h
