# Supplementary material for: Profiling mRNA, miRNA and lncRNA expression changes in endothelial cells in response to increasing doses of ionizing radiation
Source: Sci Rep. 2022 Nov 19;12:19941. doi: 10.1038/s41598-022-24051-6 (PMC9675751; doi:10.1038/s41598-022-24051-6)
Supplement: Supplementary file 3 — Supplementary Figure 3. [file 41598_2022_24051_MOESM3_ESM.pptx]

## Slide 1
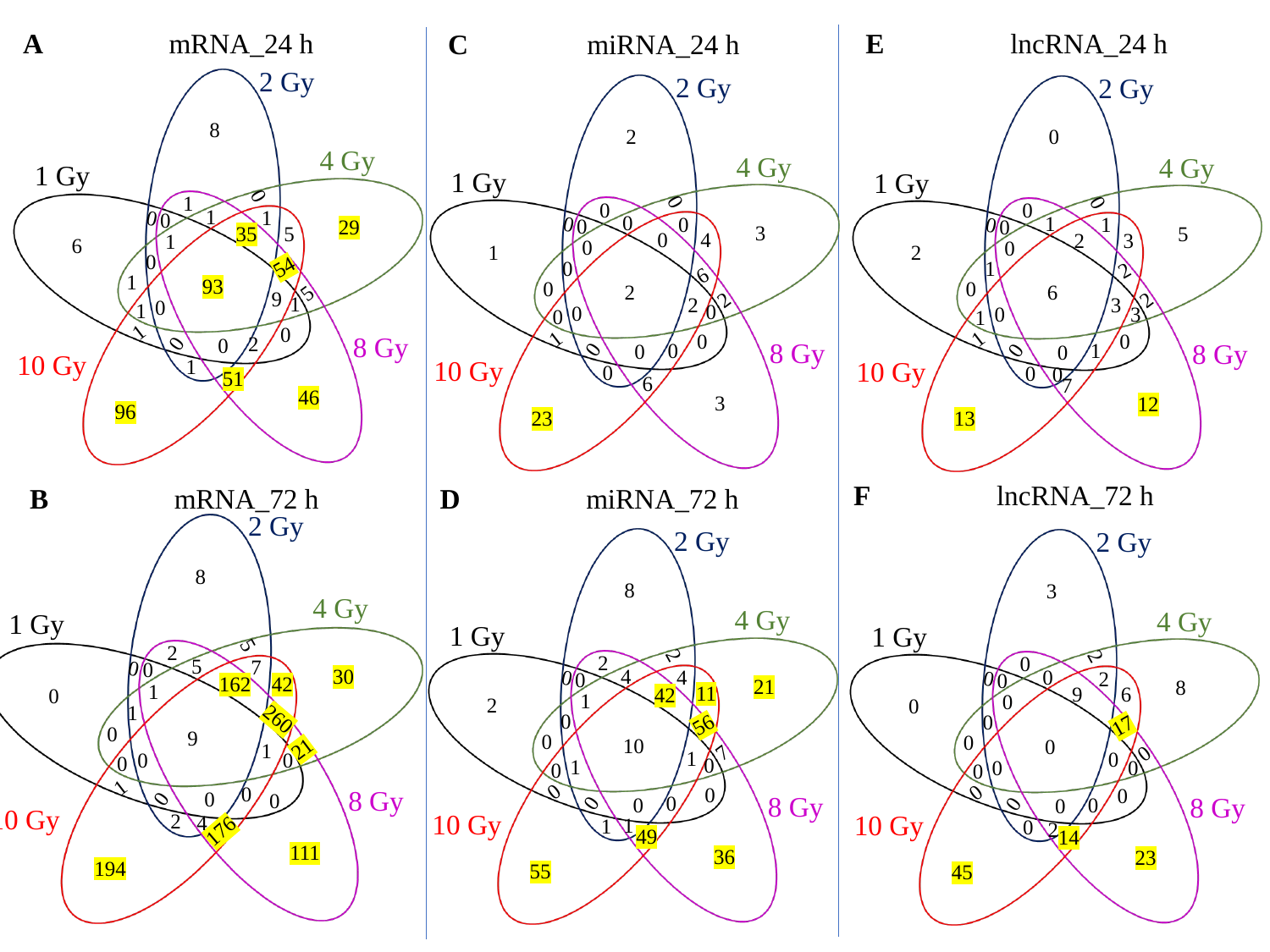

A mRNA_24 h
E lncRNA_24 h
C miRNA_24 h
2 Gy
8
4 Gy
1 Gy
0
1
0
29
35
1
6
54
93
5
9
1
8 Gy
10 Gy
46
96
1
1
0
5
0
1
0
1
1
0
0
2
0
1
51
2 Gy
2
4 Gy
1 Gy
0
0
0
3
0
0
1
6
2
2
2
0
8 Gy
10 Gy
3
23
0
0
0
4
0
0
0
0
1
0
0
0
0
0
6
2 Gy
0
4 Gy
1 Gy
0
1
0
5
2
0
2
2
6
2
3
3
8 Gy
10 Gy
12
13
0
1
0
3
1
0
0
1
1
0
0
1
0
0
7
0
F lncRNA_72 h
B mRNA_72 h
D miRNA_72 h
2 Gy
8
4 Gy
1 Gy
5
7
0
30
162
1
0
260
9
21
1
8 Gy
0
10 Gy
111
194
2 Gy
8
4 Gy
1 Gy
2
4
0
21
1
2
56
10
7
1
0
8 Gy
10 Gy
36
55
2
4
0
11
0
0
0
1
0
0
0
0
0
1
49
2 Gy
3
4 Gy
1 Gy
2
2
0
8
9
0
0
17
0
0
0
0
8 Gy
10 Gy
23
45
0
0
0
6
0
0
0
0
0
0
0
0
0
0
14
2
5
0
42
42
1
0
0
0
0
1
0
0
0
2
4
1
176
2
