# Supplementary material for: Profiling mRNA, miRNA and lncRNA expression changes in endothelial cells in response to increasing doses of ionizing radiation
Source: Sci Rep. 2022 Nov 19;12:19941. doi: 10.1038/s41598-022-24051-6 (PMC9675751; doi:10.1038/s41598-022-24051-6)
Supplement: Supplementary file 4 — Supplementary Figure 4. [file 41598_2022_24051_MOESM4_ESM.pptx]

## Slide 1
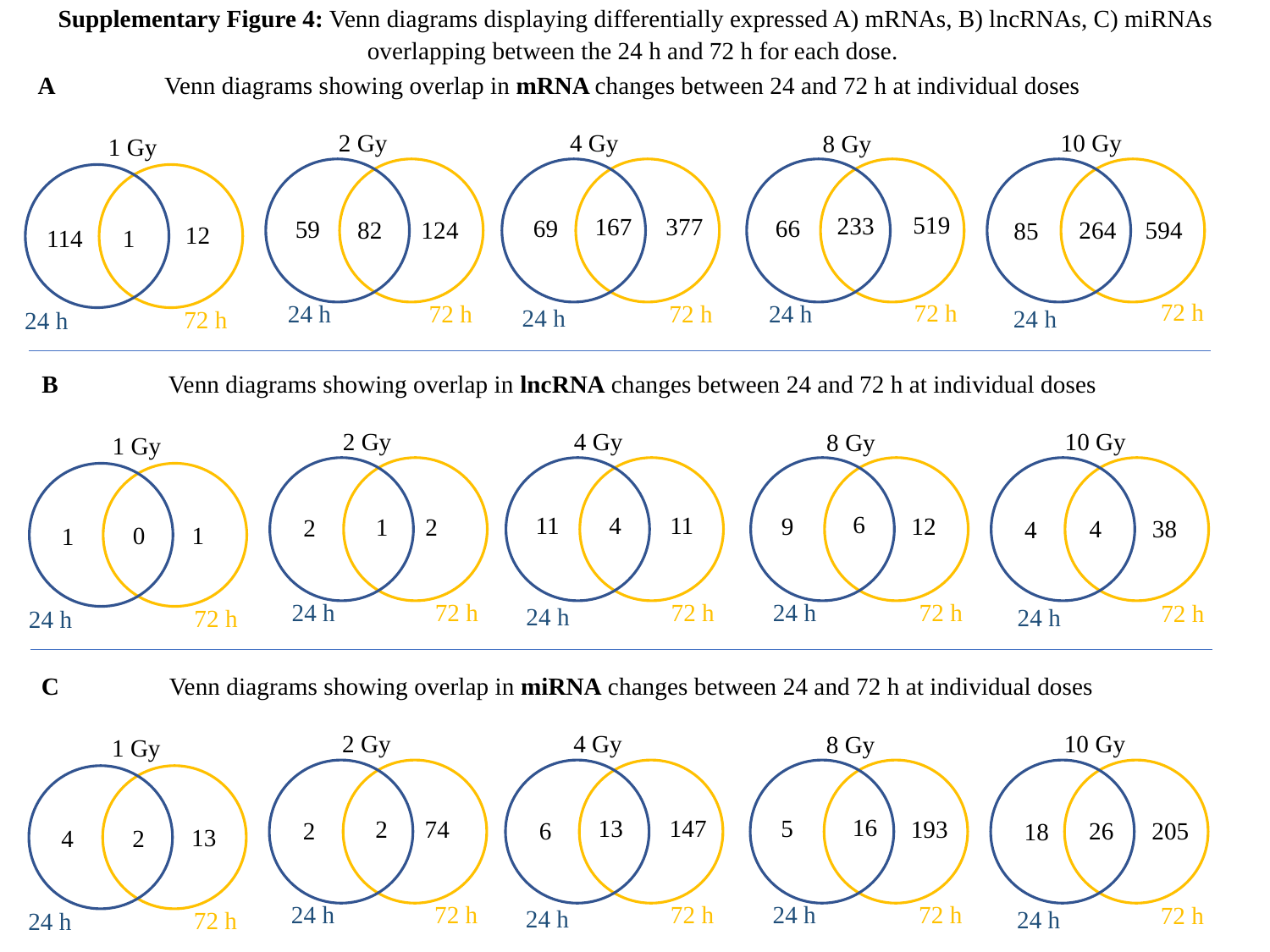

Supplementary Figure 4: Venn diagrams displaying differentially expressed A) mRNAs, B) lncRNAs, C) miRNAs overlapping between the 24 h and 72 h for each dose.
A Venn diagrams showing overlap in mRNA changes between 24 and 72 h at individual doses
2 Gy
4 Gy
10 Gy
8 Gy
1 Gy
519
233
167
377
69
66
59
264
594
82
124
85
12
1
114
72 h
72 h
24 h
72 h
24 h
72 h
24 h
24 h
72 h
24 h
B Venn diagrams showing overlap in lncRNA changes between 24 and 72 h at individual doses
2 Gy
4 Gy
10 Gy
8 Gy
1 Gy
6
11
4
11
9
12
1
2
2
4
38
4
1
0
1
72 h
24 h
72 h
72 h
24 h
72 h
24 h
24 h
72 h
24 h
C Venn diagrams showing overlap in miRNA changes between 24 and 72 h at individual doses
2 Gy
4 Gy
10 Gy
8 Gy
1 Gy
16
13
147
5
193
2
74
2
6
26
205
18
13
2
4
72 h
24 h
72 h
72 h
24 h
72 h
24 h
24 h
72 h
24 h
