# Supplementary figures and images for: Profiling mRNA, miRNA and lncRNA expression changes in endothelial cells in response to increasing doses of ionizing radiation
Source: Sci Rep. 2022 Nov 19;12:19941. doi: 10.1038/s41598-022-24051-6 (PMC9675751; doi:10.1038/s41598-022-24051-6)

A

qRT-PCR validation 24h

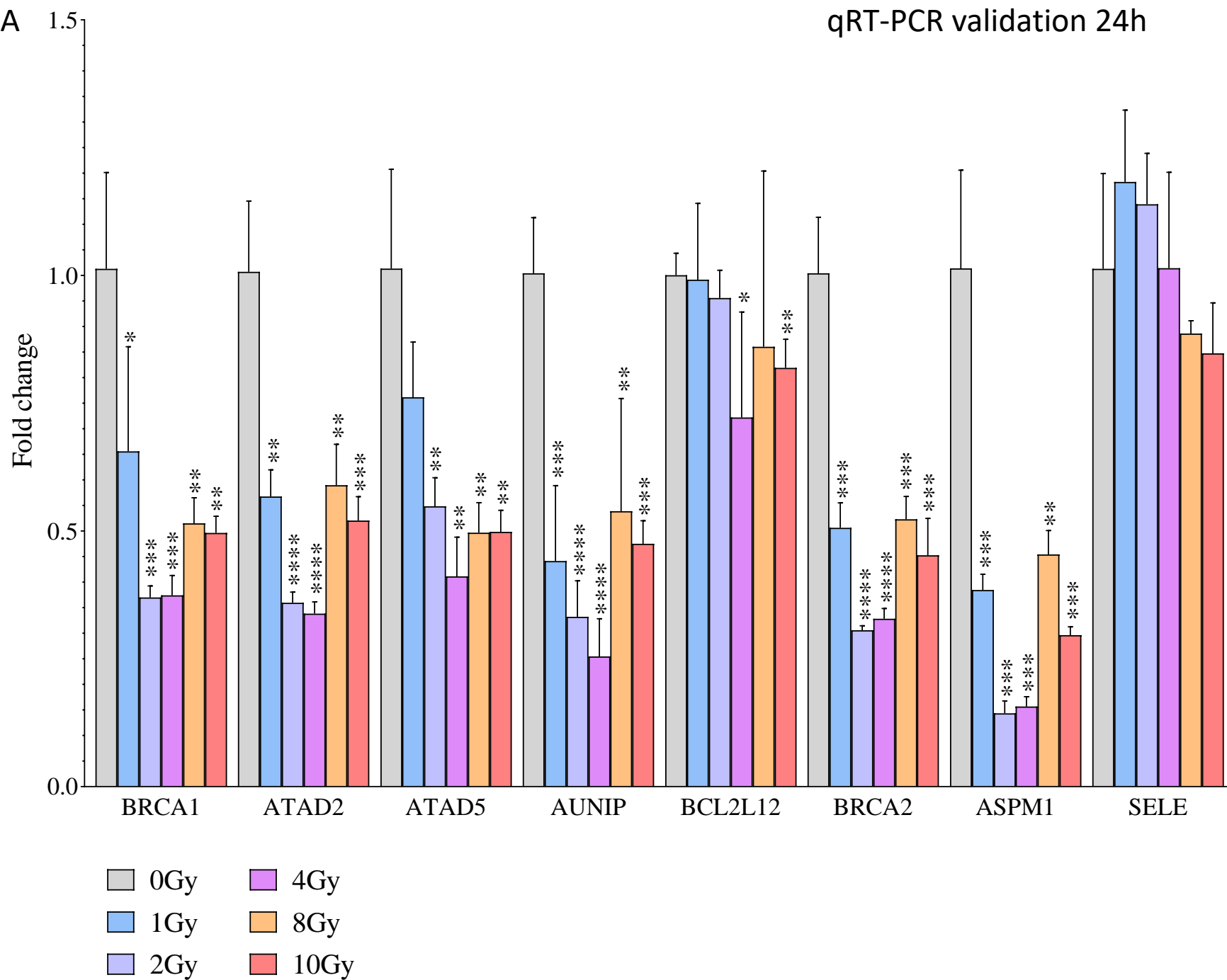

B

qRT-PCR validation 72h

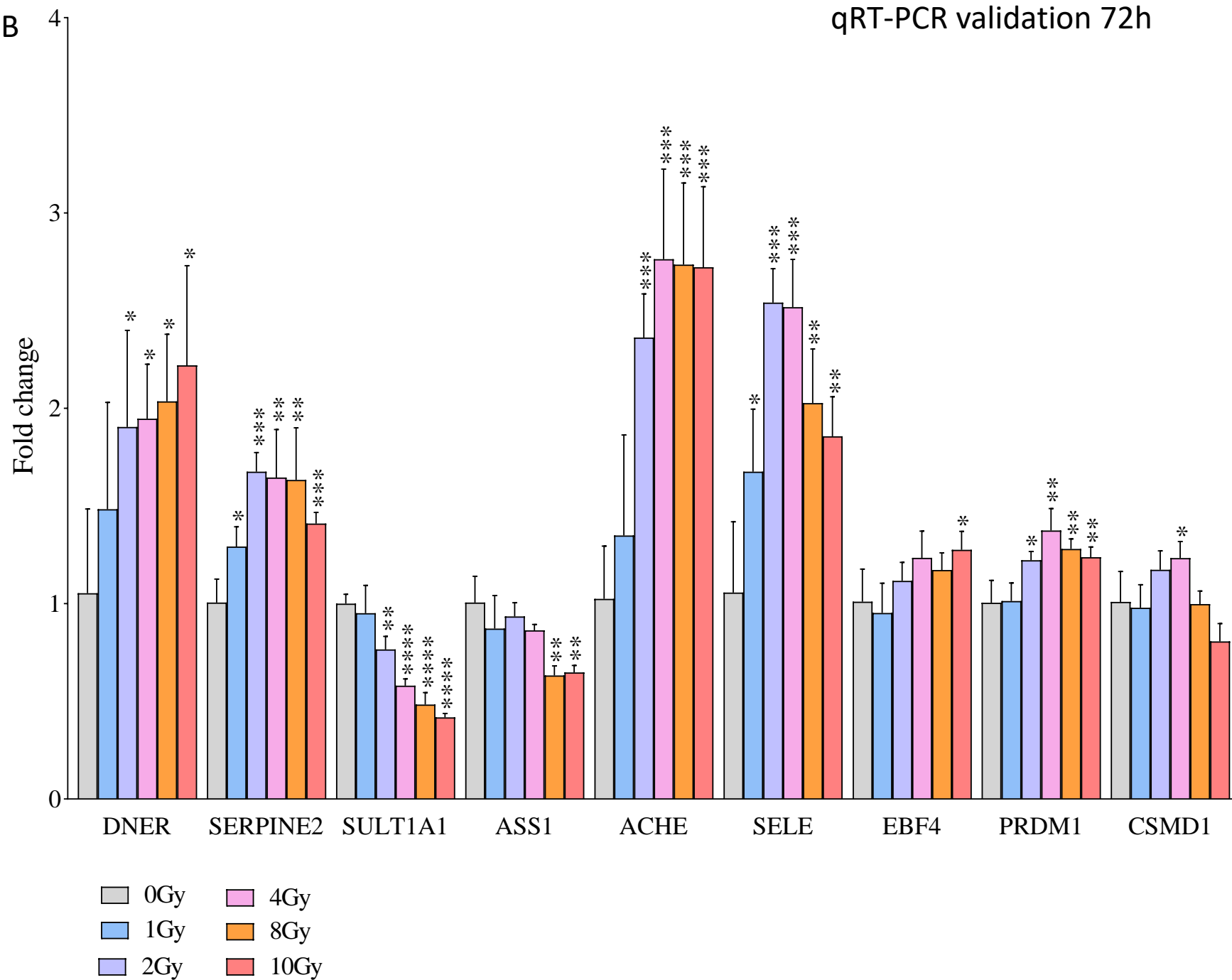

Supplement: Supplementary file 5 — Supplementary Figure 5. [file 41598_2022_24051_MOESM5_ESM.pdf]
