# Supplementary material for: Profiling mRNA, miRNA and lncRNA expression changes in endothelial cells in response to increasing doses of ionizing radiation
Source: Sci Rep. 2022 Nov 19;12:19941. doi: 10.1038/s41598-022-24051-6 (PMC9675751; doi:10.1038/s41598-022-24051-6)
Supplement: Supplementary file 6 — Supplementary Figure 6. [file 41598_2022_24051_MOESM6_ESM.pptx]

## Slide 1
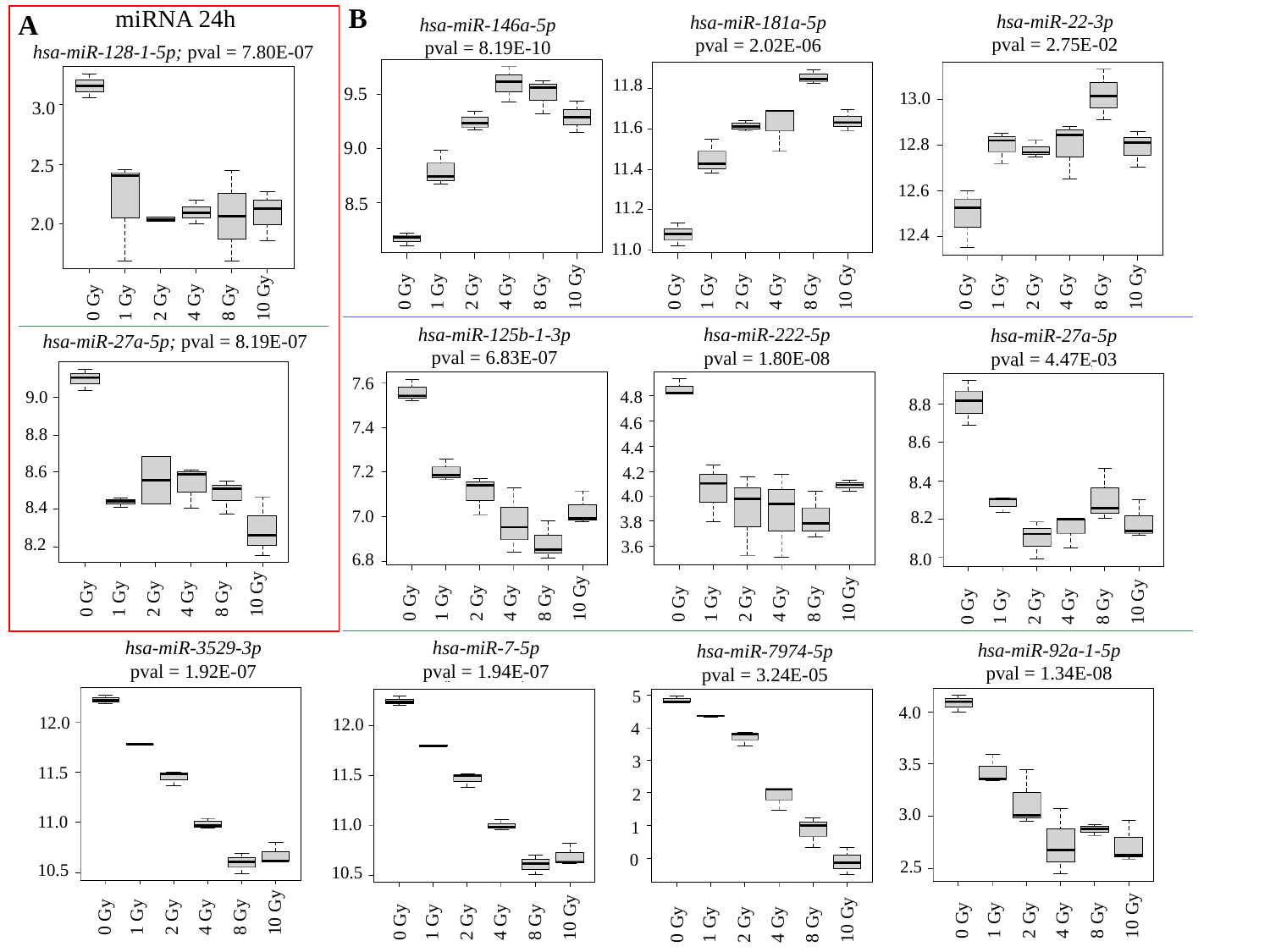

B
miRNA 24h
A
hsa-miR-22-3p
pval = 2.75E-02
hsa-miR-181a-5p
pval = 2.02E-06
hsa-miR-146a-5p
pval = 8.19E-10
hsa-miR-128-1-5p; pval = 7.80E-07
11.8
11.6
11.4
9.5
9.0
8.5
13.0
3.0
12.8
2.5
12.6
11.2
2.0
12.4
11.0
10 Gy
0 Gy
1 Gy
2 Gy
4 Gy
8 Gy
10 Gy
0 Gy
1 Gy
2 Gy
4 Gy
8 Gy
10 Gy
0 Gy
1 Gy
2 Gy
4 Gy
8 Gy
10 Gy
0 Gy
1 Gy
2 Gy
4 Gy
8 Gy
hsa-miR-125b-1-3p
pval = 6.83E-07
hsa-miR-222-5p
pval = 1.80E-08
hsa-miR-27a-5p
pval = 4.47E-03
hsa-miR-27a-5p; pval = 8.19E-07
7.6
9.0
4.8
8.8
4.6
7.4
8.8
8.6
4.4
8.6
7.2
4.2
8.4
4.0
8.4
7.0
8.2
3.8
8.2
3.6
6.8
8.0
10 Gy
0 Gy
1 Gy
2 Gy
4 Gy
8 Gy
10 Gy
0 Gy
1 Gy
2 Gy
4 Gy
8 Gy
10 Gy
0 Gy
1 Gy
2 Gy
4 Gy
8 Gy
10 Gy
0 Gy
1 Gy
2 Gy
4 Gy
8 Gy
hsa-miR-7-5p
pval = 1.94E-07
hsa-miR-3529-3p
pval = 1.92E-07
hsa-miR-92a-1-5p
pval = 1.34E-08
hsa-miR-7974-5p
pval = 3.24E-05
5
4.0
12.0
12.0
4
3
3.5
11.5
11.5
2
3.0
11.0
11.0
1
0
2.5
10.5
10.5
10 Gy
0 Gy
1 Gy
2 Gy
4 Gy
8 Gy
10 Gy
0 Gy
1 Gy
2 Gy
4 Gy
8 Gy
10 Gy
0 Gy
1 Gy
2 Gy
4 Gy
8 Gy
10 Gy
0 Gy
1 Gy
2 Gy
4 Gy
8 Gy
