# Supplementary material for: Profiling mRNA, miRNA and lncRNA expression changes in endothelial cells in response to increasing doses of ionizing radiation
Source: Sci Rep. 2022 Nov 19;12:19941. doi: 10.1038/s41598-022-24051-6 (PMC9675751; doi:10.1038/s41598-022-24051-6)
Supplement: Supplementary file 7 — Supplementary Figure 7. [file 41598_2022_24051_MOESM7_ESM.pptx]

## Slide 1
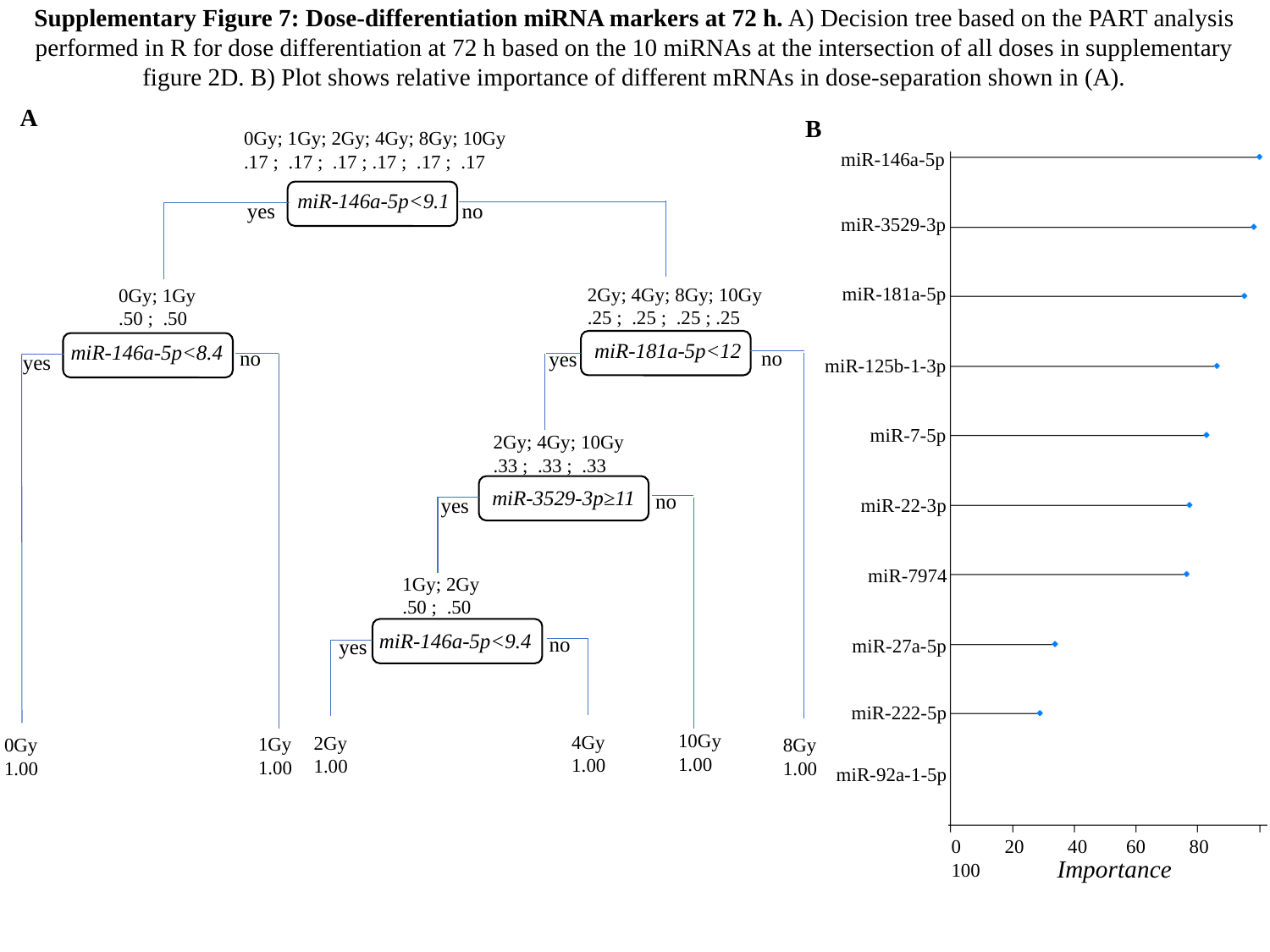

Supplementary Figure 7: Dose-differentiation miRNA markers at 72 h. A) Decision tree based on the PART analysis performed in R for dose differentiation at 72 h based on the 10 miRNAs at the intersection of all doses in supplementary figure 2D. B) Plot shows relative importance of different mRNAs in dose-separation shown in (A).
A
0Gy; 1Gy; 2Gy; 4Gy; 8Gy; 10Gy
.17 ; .17 ; .17 ; .17 ; .17 ; .17
miR-146a-5p<9.1
no
yes
2Gy; 4Gy; 8Gy; 10Gy
.25 ; .25 ; .25 ; .25
 0Gy; 1Gy
 .50 ; .50
miR-181a-5p<12
miR-146a-5p<8.4
no
no
yes
yes
2Gy; 4Gy; 10Gy
.33 ; .33 ; .33
miR-3529-3p≥11
no
yes
1Gy; 2Gy
.50 ; .50
miR-146a-5p<9.4
no
yes
10Gy
1.00
4Gy
1.00
2Gy
1.00
1Gy
1.00
0Gy
1.00
8Gy
1.00
B
miR-146a-5p
miR-3529-3p
miR-181a-5p
miR-125b-1-3p
miR-7-5p
miR-22-3p
miR-7974
miR-27a-5p
miR-222-5p
miR-92a-1-5p
0 20 40 60 80 100
Importance
