# Supplementary material for: Profiling mRNA, miRNA and lncRNA expression changes in endothelial cells in response to increasing doses of ionizing radiation
Source: Sci Rep. 2022 Nov 19;12:19941. doi: 10.1038/s41598-022-24051-6 (PMC9675751; doi:10.1038/s41598-022-24051-6)
Supplement: Supplementary file 8 — Supplementary Figure 8. [file 41598_2022_24051_MOESM8_ESM.pptx]

## Slide 1
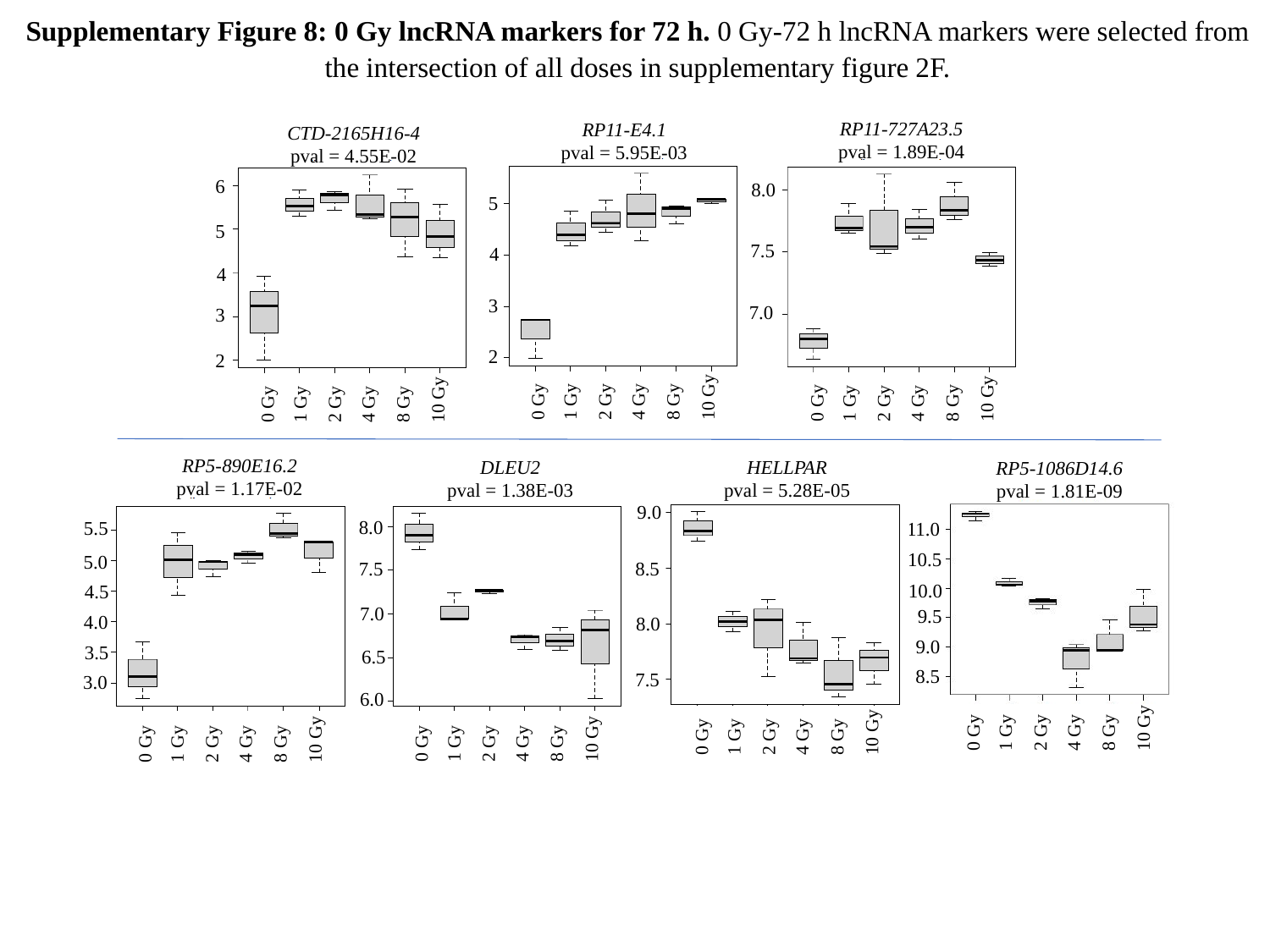

Supplementary Figure 8: 0 Gy lncRNA markers for 72 h. 0 Gy-72 h lncRNA markers were selected from the intersection of all doses in supplementary figure 2F.
RP11-727A23.5
pval = 1.89E-04
8.0
7.5
7.0
10 Gy
0 Gy
1 Gy
2 Gy
4 Gy
8 Gy
RP11-E4.1
pval = 5.95E-03
5
4
3
2
10 Gy
0 Gy
1 Gy
2 Gy
4 Gy
8 Gy
CTD-2165H16-4
pval = 4.55E-02
6
5
4
3
2
10 Gy
0 Gy
1 Gy
2 Gy
4 Gy
8 Gy
RP5-890E16.2
pval = 1.17E-02
5.5
5.0
4.5
4.0
3.5
3.0
10 Gy
0 Gy
1 Gy
2 Gy
4 Gy
8 Gy
HELLPAR
pval = 5.28E-05
9.0
8.5
8.0
7.5
10 Gy
0 Gy
1 Gy
2 Gy
4 Gy
8 Gy
DLEU2
pval = 1.38E-03
8.0
7.5
7.0
6.5
6.0
10 Gy
0 Gy
1 Gy
2 Gy
4 Gy
8 Gy
RP5-1086D14.6
pval = 1.81E-09
11.0
10.5
10.0
9.5
9.0
8.5
10 Gy
0 Gy
1 Gy
2 Gy
4 Gy
8 Gy
