# Supplementary material for: Profiling mRNA, miRNA and lncRNA expression changes in endothelial cells in response to increasing doses of ionizing radiation
Source: Sci Rep. 2022 Nov 19;12:19941. doi: 10.1038/s41598-022-24051-6 (PMC9675751; doi:10.1038/s41598-022-24051-6)
Supplement: Supplementary file 9 — Supplementary Figure 9. [file 41598_2022_24051_MOESM9_ESM.pptx]

## Slide 1
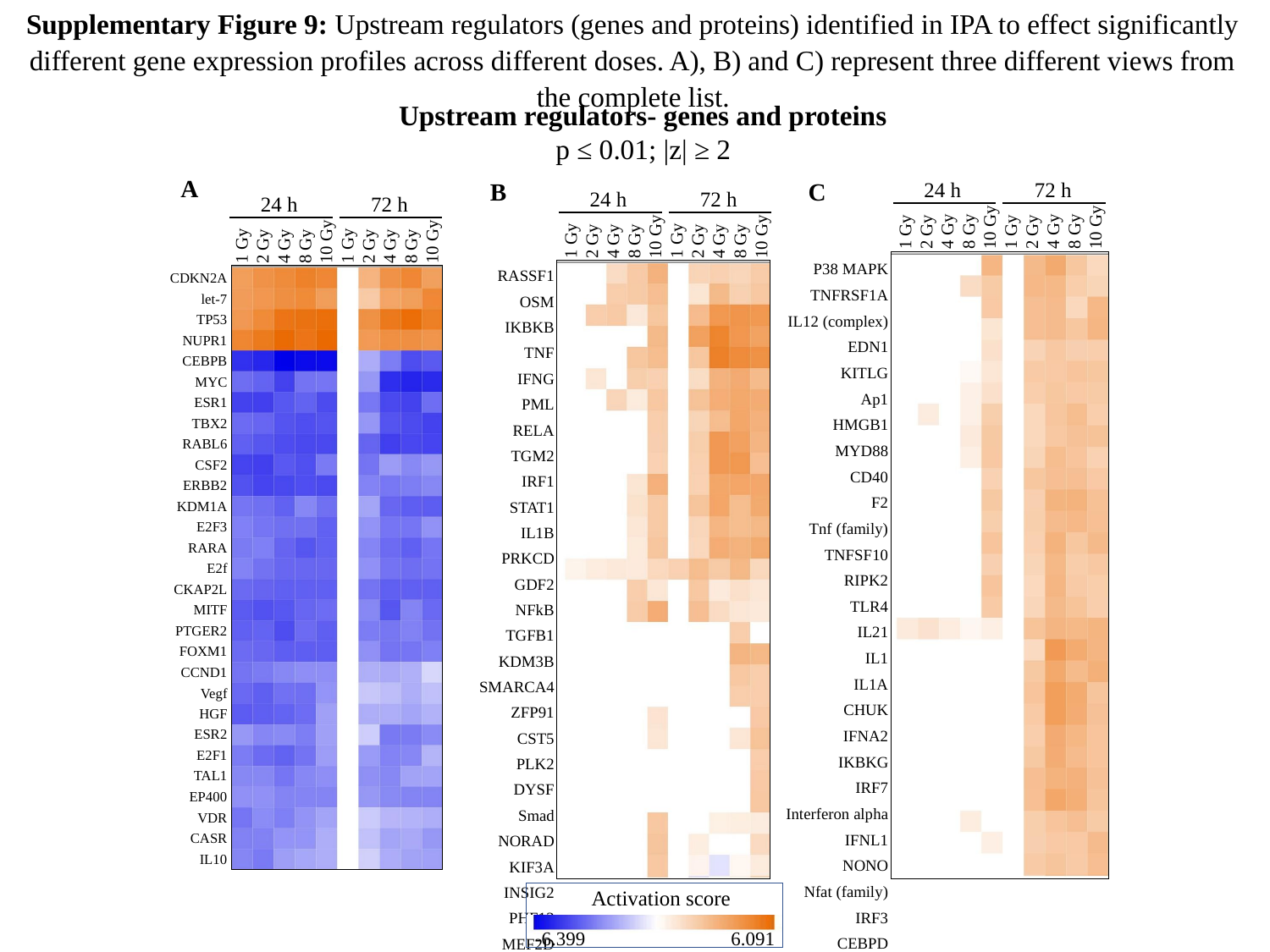

Supplementary Figure 9: Upstream regulators (genes and proteins) identified in IPA to effect significantly different gene expression profiles across different doses. A), B) and C) represent three different views from the complete list.
Upstream regulators- genes and proteins
p ≤ 0.01; |z| ≥ 2
1 Gy
2 Gy
4 Gy
8 Gy
10 Gy
1 Gy
2 Gy
4 Gy
8 Gy
10 Gy
72 h
24 h
1 Gy
2 Gy
4 Gy
8 Gy
10 Gy
1 Gy
2 Gy
4 Gy
8 Gy
10 Gy
72 h
24 h
1 Gy
2 Gy
4 Gy
8 Gy
10 Gy
1 Gy
2 Gy
4 Gy
8 Gy
10 Gy
72 h
24 h
A
C
B
| P38 MAPK |
| --- |
| TNFRSF1A |
| IL12 (complex) |
| EDN1 |
| KITLG |
| Ap1 |
| HMGB1 |
| MYD88 |
| CD40 |
| F2 |
| Tnf (family) |
| TNFSF10 |
| RIPK2 |
| TLR4 |
| IL21 |
| IL1 |
| IL1A |
| CHUK |
| IFNA2 |
| IKBKG |
| IRF7 |
| Interferon alpha |
| IFNL1 |
| NONO |
| Nfat (family) |
| IRF3 |
| CEBPD |
| GH1 |
| MIF |
| RASSF1 |
| --- |
| OSM |
| IKBKB |
| TNF |
| IFNG |
| PML |
| RELA |
| TGM2 |
| IRF1 |
| STAT1 |
| IL1B |
| PRKCD |
| GDF2 |
| NFkB |
| TGFB1 |
| KDM3B |
| SMARCA4 |
| ZFP91 |
| CST5 |
| PLK2 |
| DYSF |
| Smad |
| NORAD |
| KIF3A |
| INSIG2 |
| PHF12 |
| MEF2D |
| BHLHE40 |
| STAT6 |
| CDKN2A |
| --- |
| let-7 |
| TP53 |
| NUPR1 |
| CEBPB |
| MYC |
| ESR1 |
| TBX2 |
| RABL6 |
| CSF2 |
| ERBB2 |
| KDM1A |
| E2F3 |
| RARA |
| E2f |
| CKAP2L |
| MITF |
| PTGER2 |
| FOXM1 |
| CCND1 |
| Vegf |
| HGF |
| ESR2 |
| E2F1 |
| TAL1 |
| EP400 |
| VDR |
| CASR |
| IL10 |
Activation score
-6.399 6.091
