# Supplementary material for: Profiling mRNA, miRNA and lncRNA expression changes in endothelial cells in response to increasing doses of ionizing radiation
Source: Sci Rep. 2022 Nov 19;12:19941. doi: 10.1038/s41598-022-24051-6 (PMC9675751; doi:10.1038/s41598-022-24051-6)
Supplement: Supplementary file 10 — Supplementary Figure 10. [file 41598_2022_24051_MOESM10_ESM.pptx]

## Slide 1
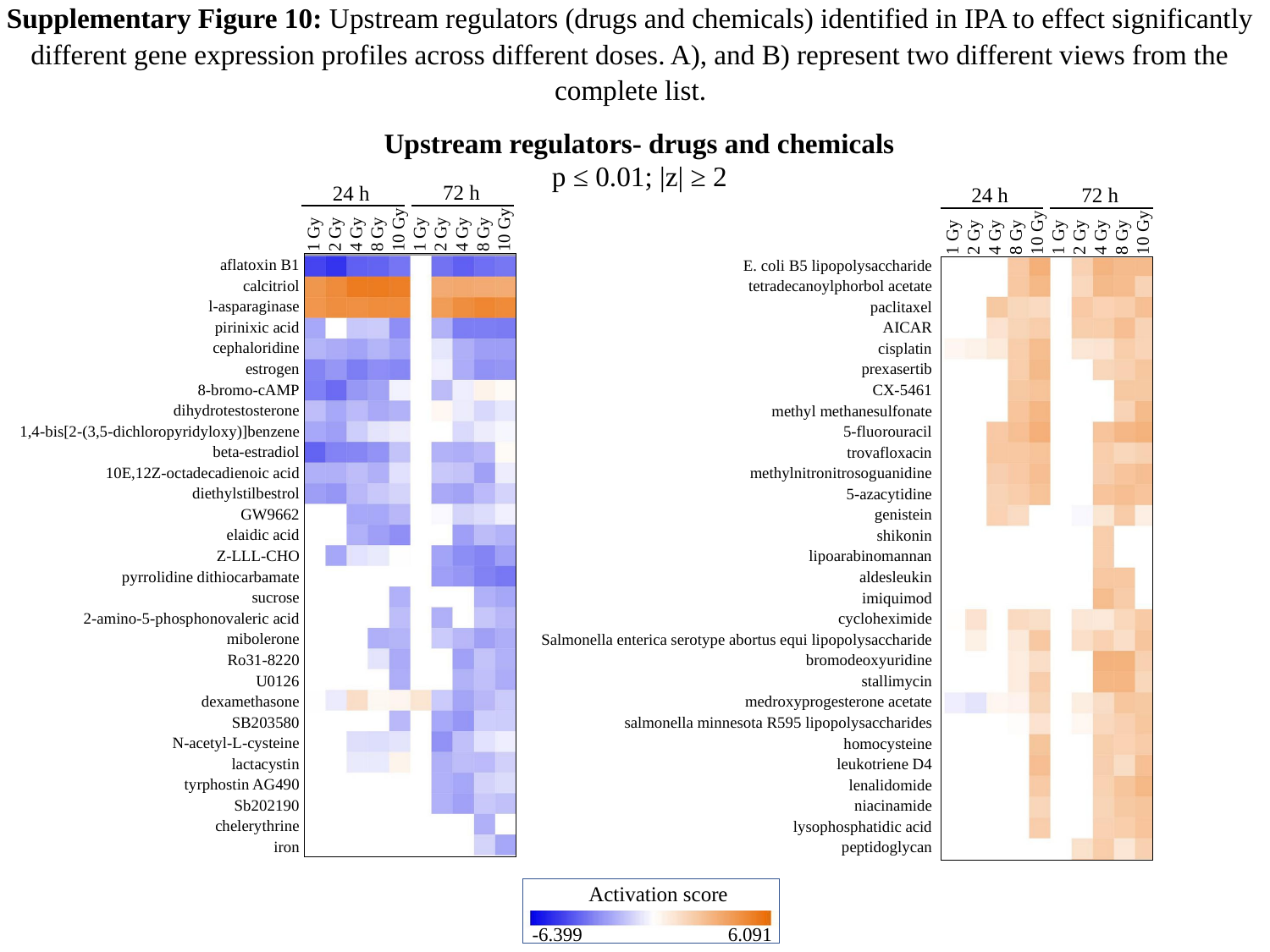

Supplementary Figure 10: Upstream regulators (drugs and chemicals) identified in IPA to effect significantly different gene expression profiles across different doses. A), and B) represent two different views from the complete list.
Upstream regulators- drugs and chemicals
p ≤ 0.01; |z| ≥ 2
1 Gy
2 Gy
4 Gy
8 Gy
10 Gy
1 Gy
2 Gy
4 Gy
8 Gy
10 Gy
72 h
24 h
1 Gy
2 Gy
4 Gy
8 Gy
10 Gy
1 Gy
2 Gy
4 Gy
8 Gy
10 Gy
72 h
24 h
| aflatoxin B1 |
| --- |
| calcitriol |
| l-asparaginase |
| pirinixic acid |
| cephaloridine |
| estrogen |
| 8-bromo-cAMP |
| dihydrotestosterone |
| 1,4-bis[2-(3,5-dichloropyridyloxy)]benzene |
| beta-estradiol |
| 10E,12Z-octadecadienoic acid |
| diethylstilbestrol |
| GW9662 |
| elaidic acid |
| Z-LLL-CHO |
| pyrrolidine dithiocarbamate |
| sucrose |
| 2-amino-5-phosphonovaleric acid |
| mibolerone |
| Ro31-8220 |
| U0126 |
| dexamethasone |
| SB203580 |
| N-acetyl-L-cysteine |
| lactacystin |
| tyrphostin AG490 |
| Sb202190 |
| chelerythrine |
| iron |
| E. coli B5 lipopolysaccharide |
| --- |
| tetradecanoylphorbol acetate |
| paclitaxel |
| AICAR |
| cisplatin |
| prexasertib |
| CX-5461 |
| methyl methanesulfonate |
| 5-fluorouracil |
| trovafloxacin |
| methylnitronitrosoguanidine |
| 5-azacytidine |
| genistein |
| shikonin |
| lipoarabinomannan |
| aldesleukin |
| imiquimod |
| cycloheximide |
| Salmonella enterica serotype abortus equi lipopolysaccharide |
| bromodeoxyuridine |
| stallimycin |
| medroxyprogesterone acetate |
| salmonella minnesota R595 lipopolysaccharides |
| homocysteine |
| leukotriene D4 |
| lenalidomide |
| niacinamide |
| lysophosphatidic acid |
| peptidoglycan |
Activation score
-6.399 6.091
