# Supplementary material for: Profiling mRNA, miRNA and lncRNA expression changes in endothelial cells in response to increasing doses of ionizing radiation
Source: Sci Rep. 2022 Nov 19;12:19941. doi: 10.1038/s41598-022-24051-6 (PMC9675751; doi:10.1038/s41598-022-24051-6)
Supplement: Supplementary file 11 — Supplementary Figure 11. [file 41598_2022_24051_MOESM11_ESM.pptx]

## Slide 1
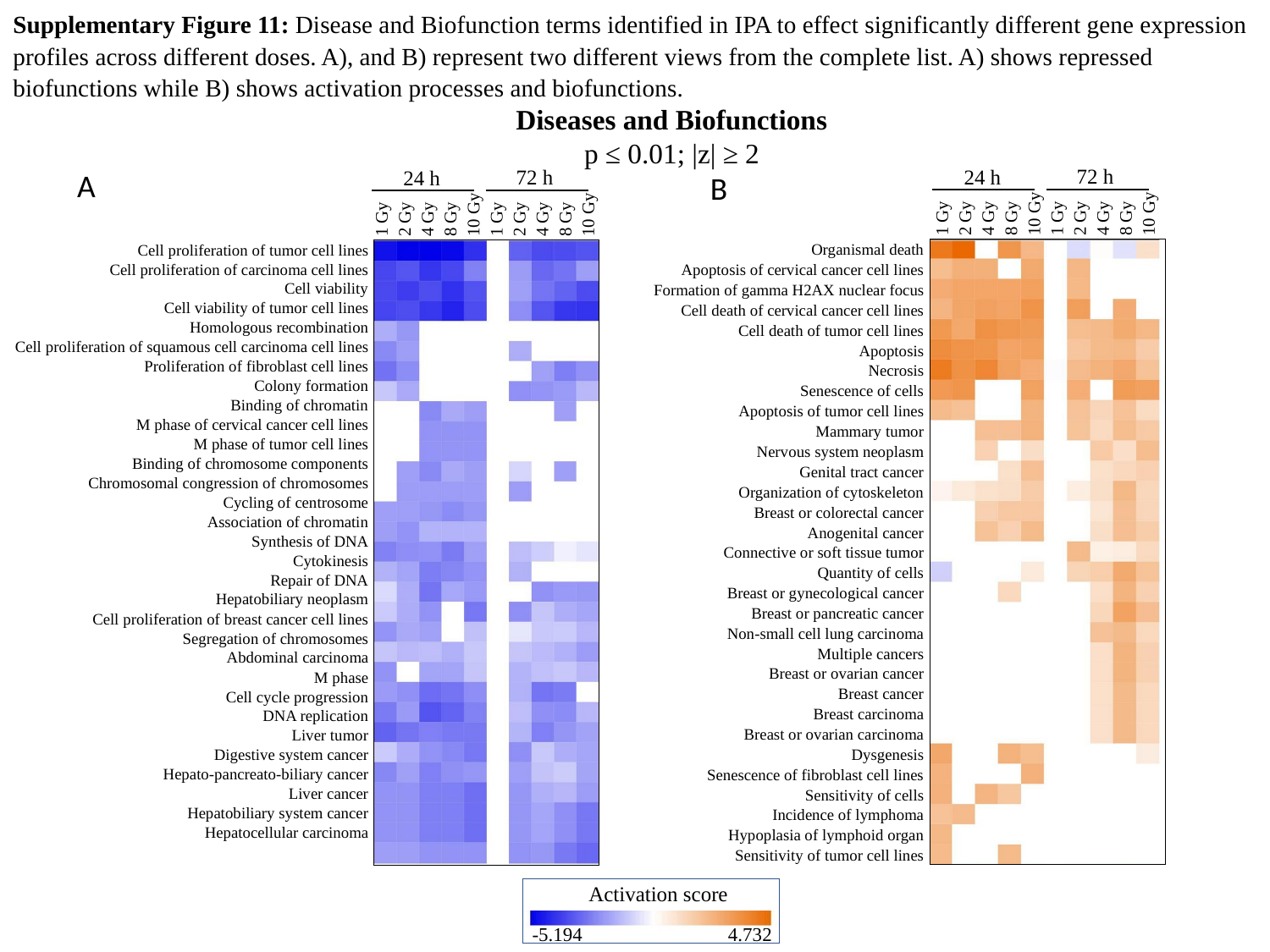

Supplementary Figure 11: Disease and Biofunction terms identified in IPA to effect significantly different gene expression profiles across different doses. A), and B) represent two different views from the complete list. A) shows repressed biofunctions while B) shows activation processes and biofunctions.
Diseases and Biofunctions
p ≤ 0.01; |z| ≥ 2
1 Gy
2 Gy
4 Gy
8 Gy
10 Gy
1 Gy
2 Gy
4 Gy
8 Gy
10 Gy
72 h
24 h
1 Gy
2 Gy
4 Gy
8 Gy
10 Gy
1 Gy
2 Gy
4 Gy
8 Gy
10 Gy
72 h
24 h
A
B
| Organismal death |
| --- |
| Apoptosis of cervical cancer cell lines |
| Formation of gamma H2AX nuclear focus |
| Cell death of cervical cancer cell lines |
| Cell death of tumor cell lines |
| Apoptosis |
| Necrosis |
| Senescence of cells |
| Apoptosis of tumor cell lines |
| Mammary tumor |
| Nervous system neoplasm |
| Genital tract cancer |
| Organization of cytoskeleton |
| Breast or colorectal cancer |
| Anogenital cancer |
| Connective or soft tissue tumor |
| Quantity of cells |
| Breast or gynecological cancer |
| Breast or pancreatic cancer |
| Non-small cell lung carcinoma |
| Multiple cancers |
| Breast or ovarian cancer |
| Breast cancer |
| Breast carcinoma |
| Breast or ovarian carcinoma |
| Dysgenesis |
| Senescence of fibroblast cell lines |
| Sensitivity of cells |
| Incidence of lymphoma |
| Hypoplasia of lymphoid organ |
| Sensitivity of tumor cell lines |
| Cell proliferation of tumor cell lines |
| --- |
| Cell proliferation of carcinoma cell lines |
| Cell viability |
| Cell viability of tumor cell lines |
| Homologous recombination |
| Cell proliferation of squamous cell carcinoma cell lines |
| Proliferation of fibroblast cell lines |
| Colony formation |
| Binding of chromatin |
| M phase of cervical cancer cell lines |
| M phase of tumor cell lines |
| Binding of chromosome components |
| Chromosomal congression of chromosomes |
| Cycling of centrosome |
| Association of chromatin |
| Synthesis of DNA |
| Cytokinesis |
| Repair of DNA |
| Hepatobiliary neoplasm |
| Cell proliferation of breast cancer cell lines |
| Segregation of chromosomes |
| Abdominal carcinoma |
| M phase |
| Cell cycle progression |
| DNA replication |
| Liver tumor |
| Digestive system cancer |
| Hepato-pancreato-biliary cancer |
| Liver cancer |
| Hepatobiliary system cancer |
| Hepatocellular carcinoma |
Activation score
-5.194 4.732
