# Supplementary material for: Profiling mRNA, miRNA and lncRNA expression changes in endothelial cells in response to increasing doses of ionizing radiation
Source: Sci Rep. 2022 Nov 19;12:19941. doi: 10.1038/s41598-022-24051-6 (PMC9675751; doi:10.1038/s41598-022-24051-6)
Supplement: Supplementary file 12 — Supplementary Figure 12. [file 41598_2022_24051_MOESM12_ESM.pdf]

Supplementary Figure 12: Gene Ontology was performed using EnrichGO package in R. Y-axis represent the top A) Molecular Function, B) Biological Process and C) Cellular Component terms across different dose time-point combinations shown along X-axis.

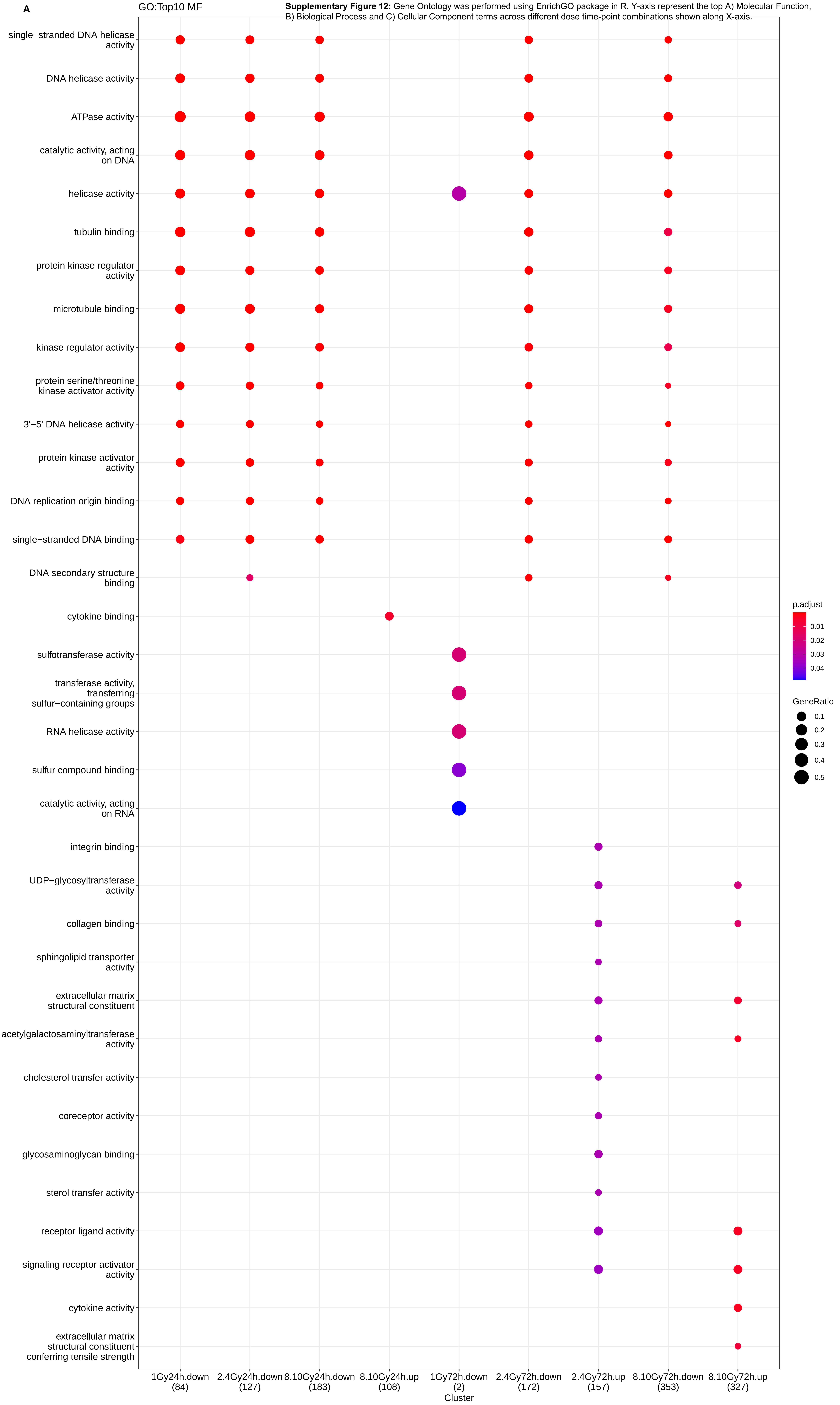

B

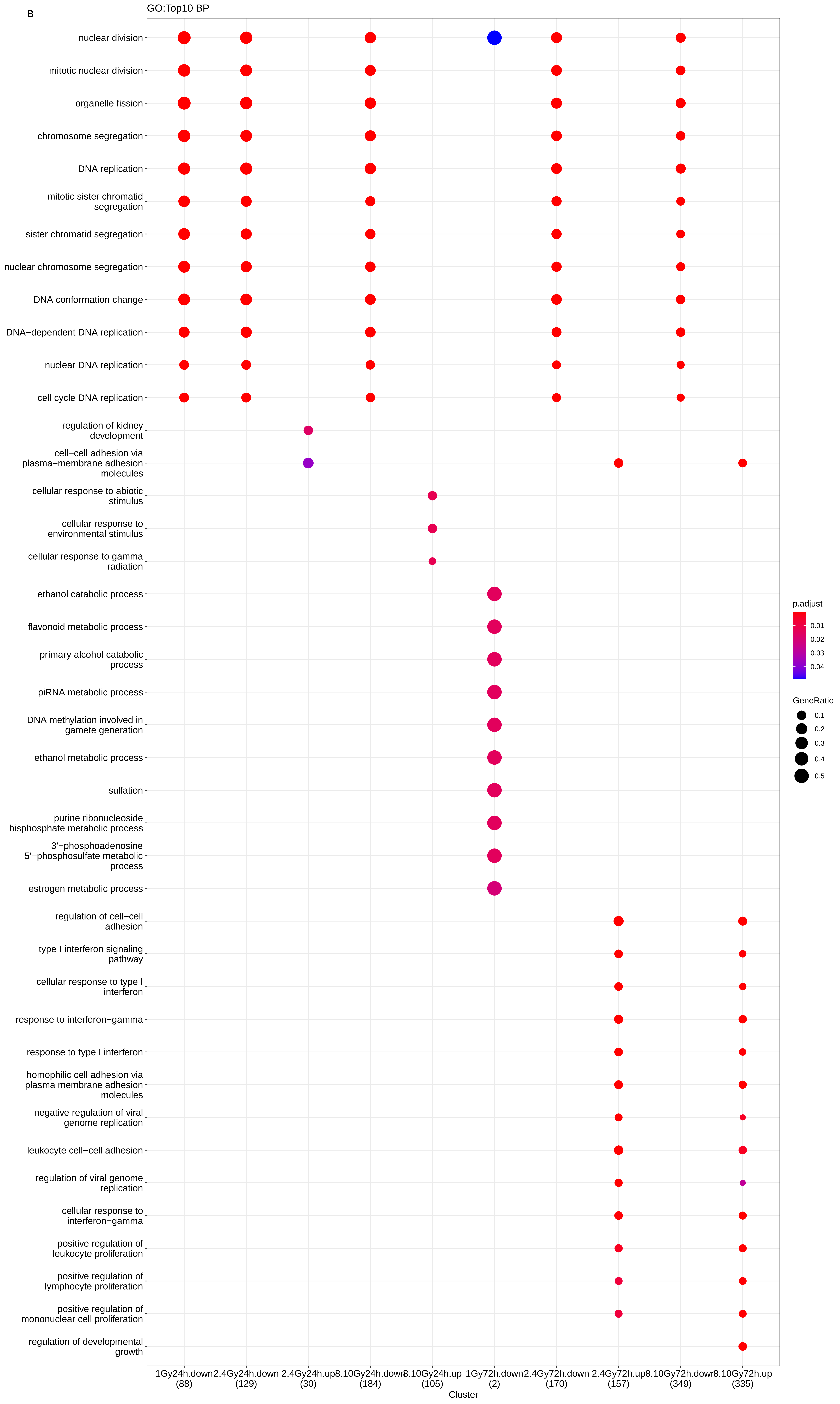
GO:Top10 BP
nuclear division
mitotic nuclear division
organelle fission
chromosome segregation
DNA replication
mitotic sister chromatid segregation
sister chromatid segregation
nuclear chromosome segregation
DNA conformation change
DNA−dependent DNA replication
nuclear DNA replication
cell cycle DNA replication
regulation of kidney development
cell−cell adhesion via plasma−membrane adhesion molecules
cellular response to abiotic stimulus
cellular response to environmental stimulus
cellular response to gamma radiation
ethanol catabolic process
flavonoid metabolic process
primary alcohol catabolic process
piRNA metabolic process
DNA methylation involved in gamete generation
ethanol metabolic process
sulfation
purine ribonucleoside bisphosphate metabolic process
3'−phosphoadenosine 5'−phosphosulfate metabolic process
estrogen metabolic process
regulation of cell−cell adhesion
type I interferon signaling pathway
cellular response to type I interferon
response to interferon−gamma
response to type I interferon
homophilic cell adhesion via plasma membrane adhesion molecules
negative regulation of viral genome replication
leukocyte cell−cell adhesion
regulation of viral genome replication
cellular response to interferon−gamma
positive regulation of leukocyte proliferation
positive regulation of lymphocyte proliferation
positive regulation of mononuclear cell proliferation
regulation of developmental growth
1Gy24h.down (88)
2.4Gy24h.down (129)
2.4Gy24h.up (30)
8.10Gy24h.down (184)
8.10Gy24h.up (105)
1Gy72h.down (2)
2.4Gy72h.down (170)
2.4Gy72h.up (157)
8.10Gy72h.down (349)
8.10Gy72h.up (335)
Cluster
p.adjust
0.01
0.02
0.03
0.04
GeneRatio
0.1
0.2
0.3
0.4
0.5

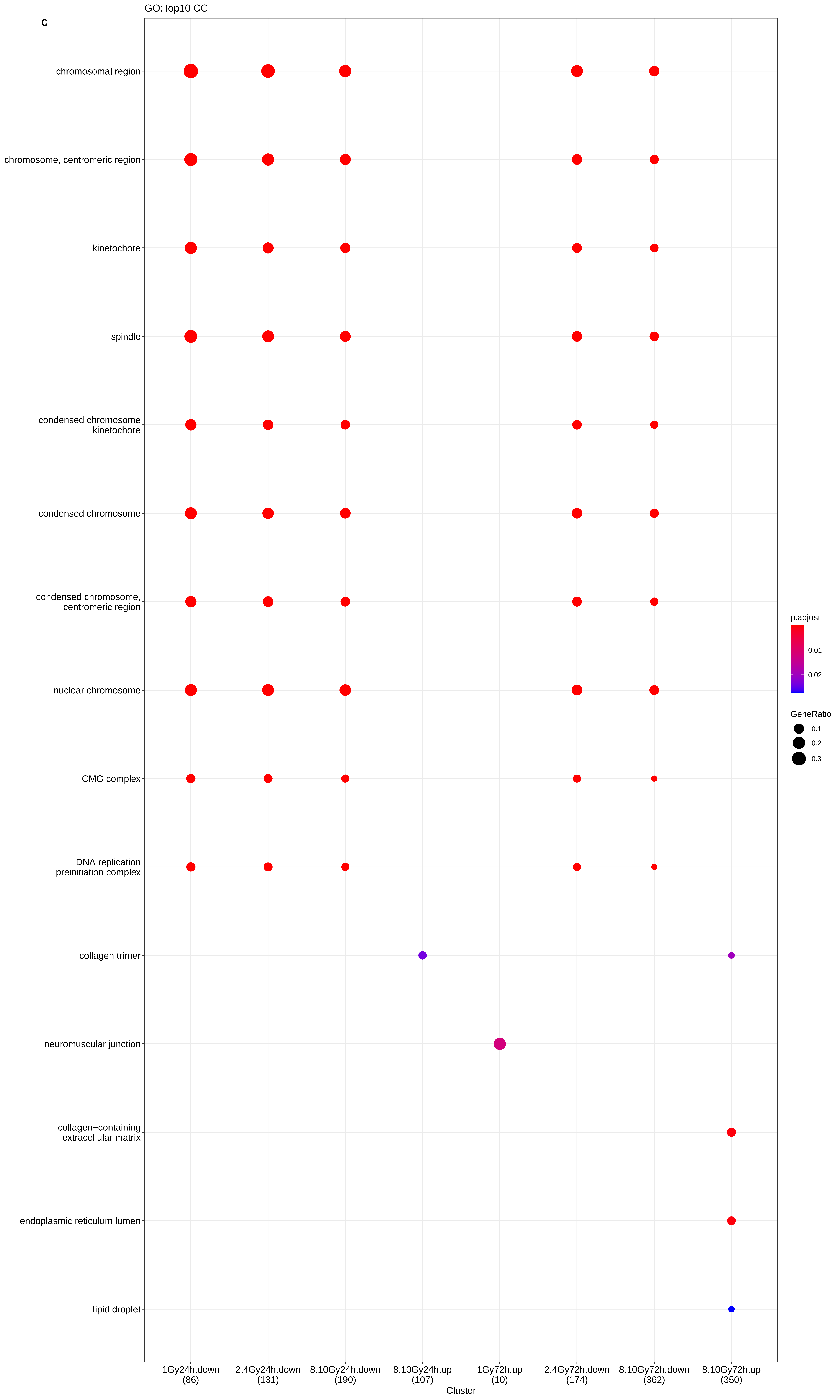
c
GO:Top10 CC
chromosomal region
chromosome, centromeric region
kinetochore
spindle
condensed chromosome kinetochore
condensed chromosome
condensed chromosome, centromeric region
nuclear chromosome
CMG complex
DNA replication preinitiation complex
collagen trimer
neuromuscular junction
collagen−containing extracellular matrix
endoplasmic reticulum lumen
lipid droplet
1Gy24h.down (86)
2.4Gy24h.down (131)
8.10Gy24h.down (190)
8.10Gy24h.up (107)
1Gy72h.up (10)
2.4Gy72h.down (174)
8.10Gy72h.down (362)
8.10Gy72h.up (350)
Cluster
p.adjust
0.01
0.02
GeneRatio
0.1
0.2
0.3
