# Supplementary material for: Profiling mRNA, miRNA and lncRNA expression changes in endothelial cells in response to increasing doses of ionizing radiation
Source: Sci Rep. 2022 Nov 19;12:19941. doi: 10.1038/s41598-022-24051-6 (PMC9675751; doi:10.1038/s41598-022-24051-6)
Supplement: Supplementary file 13 — Supplementary Legends. [file 41598_2022_24051_MOESM13_ESM.docx]

**Supplementary Figure 1:** Principal component analysis (PCA) plots were generated in R using package on the normalized read counts for A) mRNA, B) lncRNA, and C) miRNA data. Different dose-time point combinations are denoted by condition and different colors to visualize clustering of similar samples. mRNA (A), lncRNA (B) and miRNA (C) data plots show distinct 24 h and 72 h areas. Each dose-time point sample tends to cluster with its same dose-time point sample or similar dose time point sample for all doses for mRNA and lncRNA. miRNA (C) plot showing overlap of 1 Gy, 2 Gy and 4 Gy at 24 h but all doses were separately clustered at the 72 h. This could result from lower number of miRNAs expressed at 24 in lower dosed samples and regulated in response to radiation which is indeed the case.

**Supplementary Figure 2:** Heatmaps for differentially expressed A) mRNAs, B) lncRNAs and C) miRNAs based on ANOVA (FDR<0.05)

**Supplementary Figure 3:** Venn diagrams displaying the overlapping and intersecting differentially expressed mRNAs, miRNAs and lncRNAs at the 24 h and 72 h time-points. Venn diagrams were generated on interactiVenn.net

**Supplementary Figure 4:** Venn diagrams displaying differentially expressed A) mRNAs, B) lncRNAs, C) miRNAs overlapping between the 24 h and 72 h for each dose. Venn diagrams were generated on interactiVenn.net

**Supplementary Figure 5:** qRT-PCR results for validating RNAseq findings. Y-axis represents fold change. Bars denote average fold change and error bars represent standard deviation (n=4). Mean CT values across 0 Gy samples was considered as control and all fold changes were calculated in comparison to that. GAPDH was used as the normalizing gene. P-values were calculated using student’s unpaired t-test. *, **, ***, and **** represent p<0.05, p<0.01, p<0.001 and p<0.0001 respectively. Different dose points are shown by different colored bars. A) Genes differentially expressed at 24 h, and B) Genes differentially expressed at the 72 h.

**Supplementary Figure 6: 0** **Gy miRNA markers for A) 24 h and B) 72 h.** 0 Gy-24 h miRNA markers were selected from the intersection of all doses in supplementary figure 2C while 0 Gy- 72 h markers were selected from the intersection of all doses in supplementary figure 2D.

**Supplementary Figure 7:** **Dose-differentiation miRNA markers at 72 h.** A) Decision tree based on the PART analysis performed in R for dose differentiation at 72 h based on the 10 miRNAs at the intersection of all doses in supplementary figure 2D. B) Plot shows relative importance of different mRNAs in dose-separation shown in (A).

**Supplementary Figure 8:** **0 Gy lncRNA markers for 72 h.** 0 Gy-72 h lncRNA markers were selected from the intersection of all doses in supplementary figure 2F.

**Supplementary Figure 9:** Upstream regulators (genes and proteins) identified in IPA to effect significantly different gene expression profiles across different doses. A), B) and C) represent three different views from the complete list.

**Supplementary Figure 10:** Upstream regulators (drugs and chemicals) identified in IPA to effect significantly different gene expression profiles across different doses. A), and B) represent two different views from the complete list.

**Supplementary Figure 11:** Disease and Biofunction terms identified in IPA to effect significantly different gene expression profiles across different doses. A), and B) represent two different views from the complete list. A) shows repressed biofunctions while B) shows activation processes and biofunctions.

**Supplementary Figure 12:** Gene Ontology was performed using EnrichGO package in R. Y-axis represent the top A) Molecular Function, B) Biological Process and C) Cellular Component terms across different dose time-point combinations shown along X-axis.
